# Assessing Relapse in Multiple Sclerosis Questionnaire: Results of a Pilot Study

**DOI:** 10.1155/2013/470476

**Published:** 2013-05-26

**Authors:** Amy Perrin Ross, Alona Williamson, Jennifer Smrtka, Tracy Flemming Tracy, Carol Saunders, Constance Easterling, John Niewoehner, Nicole Mutschler

**Affiliations:** ^1^Department of Neurosciences, Loyola University Chicago, 2160 South First Avenue, Maywood, IL 60153, USA; ^2^Neurology Center of Fairfax, 3020 Hamaker Court, Suite 400, Fairfax, VA 22031, USA; ^3^South Florida Neurology Associates, 1601 Clint Moore Road, Suite 120, Boca Raton, FL 33487, USA; ^4^Tanner Center and Foundation for MS, 509 Brookwood Boulevard, Suite 101, Birmingham, AL 35209, USA; ^5^Neurology Center, 3907 Waring Road, Suite 2, Oceanside, CA 92056, USA; ^6^Multiple Sclerosis Care Center of Neurological Services, 3849 Oakwater Circle, Orlando, FL 38206, USA; ^7^Questcor Pharmaceuticals, 26118 Research Road, Hayward, CA 94545, USA

## Abstract

There is need for a brief but comprehensive objective assessment tool to help clinicians evaluate relapse symptoms in patients with multiple sclerosis (MS) and their impact on daily functioning, as well as response to treatment. The 2-part Assessing Relapse in Multiple Sclerosis (ARMS) questionnaire was developed to achieve these aims. Part 1 consists of 7 questions that evaluate relapse symptoms, impact on activities of daily living (ADL), overall functioning, and response to treatment for previous relapses. Part 2 consists of 7 questions that evaluate treatment response in terms of symptom relief, functioning, and tolerability. The ARMS questionnaire has been evaluated in 103 patients with MS. The most commonly reported relapse symptoms were numbness/tingling (67%), fatigue (58%), and leg/foot weakness (55%). Over half of patients reported that ADL or overall functioning were affected very much (47%) or severely (11%) by relapses. Prescribed treatments for relapses included intravenous and/or oral corticosteroids (87%) and adrenocorticotropic hormone (13%). Nearly half of patients reported that their symptoms were very much (33%) or completely resolved (16%) following treatment. The most commonly reported adverse events were sleep disturbance (45%), mood changes (33%), weight gain (29%), and increased appetite (26%). Systematic assessment of relapses and response to relapse treatment may help clinicians to optimize outcomes for MS patients.

## 1. Introduction

Multiple sclerosis (MS), particularly relapsing remitting MS (RRMS), the most common form of the disease, is characterized by relapses [1, 2]. These events vary considerably with respect to both the type and severity of symptoms [3, 4]. Relapses are cardinal events for patients with MS. They are associated with significant disability and increased cost of care; and they may result in residual deficits after resolution of the acute event [1–5]. Although relapses can be expected to resolve over time without intervention [2], treatment with high-dose corticosteroids or adrenocorticotropic hormone (ACTH) (e.g., H.P. Acthar Gel, repository corticotropin injection; Questcor Pharmaceuticals, Inc., Hayward, CA, USA) can shorten the time to recovery [6].

 Assessment tools, such as the Expanded Disability Status Scale (EDSS) [7] and Multiple Sclerosis Functional Composite (MSFC) [8], have been developed to evaluate the status of patients and disease progression in MS patients not experiencing relapses. There is also need for a tool to facilitate identification of relapses, determination of symptom severity, and evaluation of relapse treatment efficacy. Failure to identify exacerbations may delay a needed switch or intensification in disease-modifying therapy, a particularly important issue given the increasing number of options for disease-modifying therapy. Once a relapse has been identified, an instrument to evaluate the presence and severity of relapse symptoms and resulting functional impairment can aid clinicians; considering the highly variable presentation of relapses [3, 4], reliance on nonspecific assessments or spontaneous patient reports may result in failure to identify subtle symptoms and/or functional impairment that warrant intervention. Equally important is a thorough postrelapse assessment to determine whether symptoms have fully resolved and patients are able to function as well as they did before the relapse and to identify adverse events. Recent data from the North American Research Committee on Multiple Sclerosis (NARCOMS) registry show that nearly one-third of patients whose relapses were treated with high-dose corticosteroids felt that their treatment did not improve or worsened their relapse symptoms [9]. This illustrates the need for adequate followup and highlights the importance of evaluating patient satisfaction. Recognizing suboptimal response, tolerability issues, and/or lack of patient satisfaction with treatment is important not only to determine whether further intervention is necessary for a given relapse, but also to guide treatment decisions for future relapses.

The Assessing Relapse in Multiple Sclerosis (ARMS) questionnaire is a 2-part patient self-report assessment tool that was developed by a panel of expert MS nurses and is specifically designed for evaluating relapses and responses to relapse treatment [10]. Part 1 intended utilization is to evaluate patients when they present with a new relapse. It consists of 7 questions designed to evaluate relapse symptoms, the impact of symptoms on daily activities and overall functioning, and response to past treatments for previous relapses (for consideration in treatment selection). Part 2 intended utilization is to evaluate patients after relapse treatment (approximately 1 month after the initial assessment). It consists of 7 questions to evaluate the patient's perceived treatment response in terms of symptom relief, functioning, and tolerability. This paper reports the results of a pilot study designed to assess the psychometric properties of the ARMS questionnaire.

## 2. Methods

### 2.1. Study Design

This phase 4, pilot, cross-sectional descriptive/exploratory study was conducted at 5 clinical practice sites in the United States to assess the psychometric properties of the ARMS questionnaire in adult patients with MS experiencing a relapse (relapse was determined by the investigator; specific criteria were not defined by the study protocol). 

The ARMS questionnaire (see Appendix 1 of the Supplementary Material available online at http://dx.doi.org/10.1155/2013/470476) is a 2-part, 2-page survey, with each part comprising 7 questions. Part 1 was completed when a patient presented with a new MS relapse to evaluate the patient's relapse symptoms and how the symptoms affect activities of daily living (ADL) and overall function, as well as the patient's response to past treatments for previous relapses, as a means of guiding treatment selection (the choice of relapse treatment was entirely at the discretion of the treating clinician and did not affect patient eligibility). Part 2 was completed 1 month (±1 week) after initiation of treatment for relapse to evaluate the treatment response in terms of symptom relief and functioning, as well as tolerability. Each part of the questionnaire was completed by the patient or by the investigator (after verbal questioning of the patient). Questionnaires could be completed in the office or via telephone.

### 2.2. Patients

The study included patients aged 18 years or older at the time of participation with a confirmed MS relapse, as determined by the investigator, and willingness to comply with all procedures and assessments. Patients or their designated representative provided informed written consent. Patients with pseudorelapse or any other condition that, in the opinion of the investigator, would not allow proper completion of the study were excluded.

### 2.3. Data Analysis

Demographics and baseline characteristics were summarized using descriptive statistics. Responses to each item in Parts 1 and 2 of the ARMS questionnaire were summarized using descriptive statistics. Responses to items in Part 2 were summarized for the overall population and stratified by relapse treatment; post-hoc analyses evaluated differences between treatment groups.

A total composite score (TCS) was determined, using the three inter-related questions regarding response to relapse treatment. In Part 2, for example, questions 4 (symptom improvement), 5 (ADL), and 6 (return to previous state of health (RSH)) were evaluated. The TCS was calculated as the sum of the scores for these items. For this analysis, the score for the ADL item was calculated as 10 minus the value of the rating indicated by the patient, such that higher score values and greater positive changes from baseline indicate better functioning/improvement. Thus, for the TCS, the sum of the responses of the three questions has a range of 0 to 30, with higher scores indicating greater improvement/better functioning. The TCS was summarized descriptively. The distribution of the mean TCS was computed, and the 95% two-sided confidence interval for the mean TCS was computed based on Students *t*-distribution. Cronbach's *α* [11] was used to estimate the internal reliability and consistency of the three interrelated questions (Part 2, questions 4, 5, and 6) and the TCS.

Two questions (Part 1, question 3 and Part 2, question 5) both specifically refer to ADL; the change in ADL was estimated based on these two questions; the score for each item was calculated as 10 minus the value of the rating indicated by the patient, such that higher score values and greater positive changes from baseline indicate better functioning/improvement. Two other questions (Part 1, question 6 and Part 2, question 6) both specifically refer to return to previous state of health (RSH); the change in RSH was estimated based on these two questions (using the value of the rating indicated by the patient), with higher scores indicating a more complete return to previous state of health. Changes in ADL and RSH scores were summarized descriptively. The distribution of the mean change in ADL and RSH scores was also computed. The 95% two-sided confidence interval for the mean change in ADL and RSH scores was computed based on Students *t*-distribution. The internal consistency of the ADL and RSH questions in Part 1 and, separately, the ADL and RSH questions in Part 2, was examined using the Pearson correlation.

An additional composite score (partial composite score (PCS)) was computed based on the sum of the ADL and RSH questions. The PCS was computed separately for Part 1 (new relapse) and Part 2 (after relapse treatment) and summarized descriptively; again, the scores for the ADL items were calculated as 10 minus the rating provided by the patient, such that the sum of the item scores has a range of 0 to 20, with higher scores indicating better functioning/greater improvement. The distributions of the PCS mean composite scores were also computed, and the 95% two-sided confidence interval for the mean PCS was computed based on Students *t*-distribution. The change in the PCS was also computed and summarized, and the mean change was evaluated in the same manner as the PCS scores.

## 3. Results

### 3.1. Patients

The study included 103 patients. A summary of their demographic and clinical characteristics is provided in Table 1. Most questionnaires were completed in the office (93%) and were completed by the patient (86%).

### 3.2. Part 1—New Relapse Assessment

#### 3.2.1. Characteristics of Current Relapse

**Figure 1:**
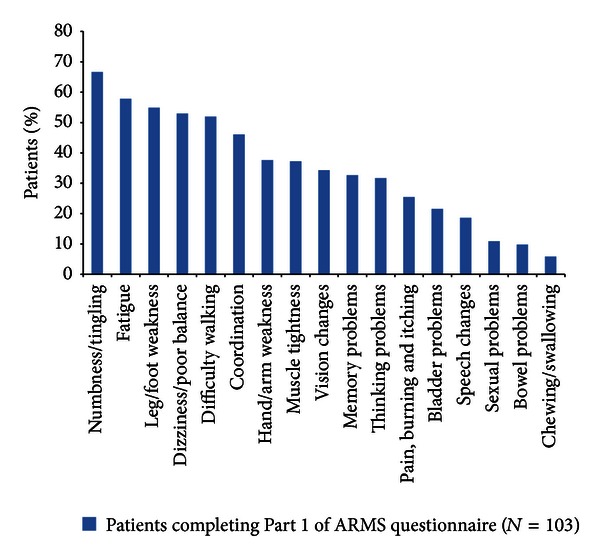
New or worsening symptoms of current (new) relapse. ARMS: Assessing Relapse in Multiple Sclerosis.

Characteristics of the current relapse are summarized in Table 2 and Figure 1. The most common new or worsening symptoms were numbness/tingling (67%), fatigue (58%), and leg/foot weakness (55%). Questionnaire results indicated that 67% of patients had their symptoms start ≥8 days prior to completion of Part 1 of the ARMS and that relapses very much or severely affected ADL in 58% of patients.

#### 3.2.2. Treatment of Previous Relapse

**Figure 2:**
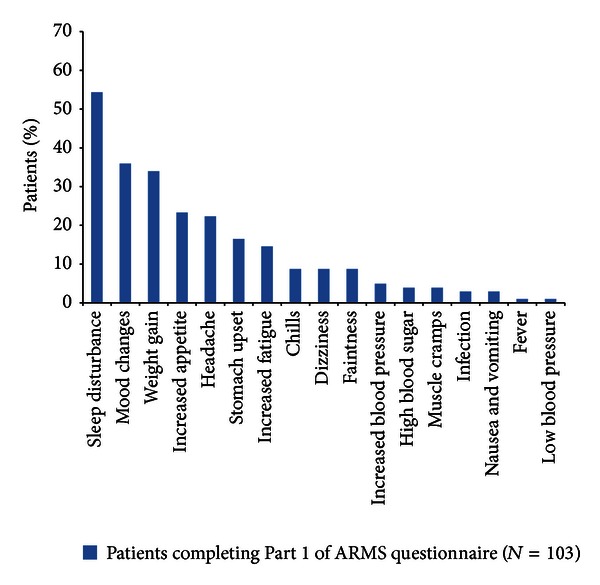
Adverse events associated with last relapse treatment. ARMS: Assessing Relapse in Multiple Sclerosis.

Most patients (82%) were treated with intravenous (IV) and/or oral corticosteroids for their last relapse; and 72% of all patients indicated that they were very much improved or completely returned to their baseline state of health after treatment for their last relapse (Table 3). The adverse events most often associated with previous relapse treatments were sleep disturbance (54%), mood changes (36%), weight gain (34%), increased appetite (23%), and headache (22%) (Figure 2). 

### 3.3. Part 2—After Relapse Treatment

All 103 patients who completed Part 1 of the ARMS also completed Part 2. The majority of follow-up assessments were conducted by phone (74%), and the majority of questionnaires were completed by office staff (79%).

The majority of patients were treated with corticosteroids for their current relapse (87%). Adrenocorticotropic hormone (ACTH) was the only other treatment reported (13%) (Table 4). Nearly all patients (97%) completed their prescribed treatment; the mean (SD) time from completion of treatment until completion of Part 2 of the questionnaire was 28.2 (9.7) days (Table 4). Nearly one-half of patients (49%) reported that their symptoms were very much improved or completely resolved following treatment; 49% reported that their ADL were affected not at all or a little; and 43% reported that they returned very much or completely to their baseline state of health (Table 4).

**Figure 3:**
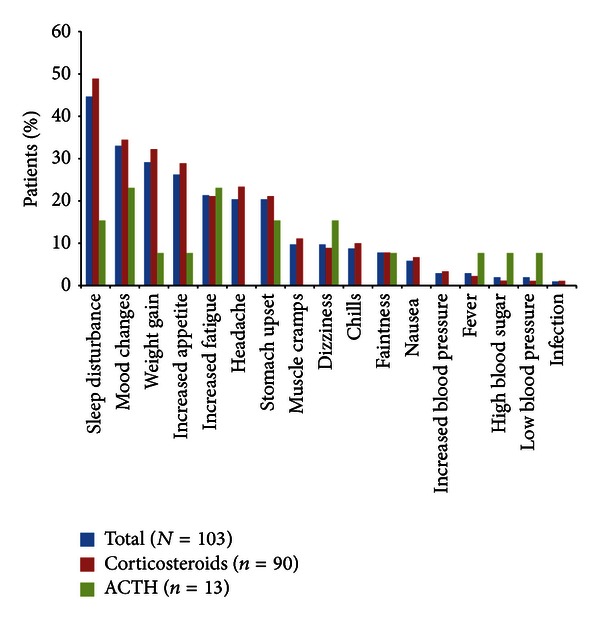
Adverse events with treatment for current relapse. ACTH: adrenocorticotropic hormone; ARMS: Assessing Relapse in Multiple Sclerosis.

The most common adverse events reported were sleep disturbance (overall incidence 45%), mood changes (33%), weight gain (29%), increased appetite (26%), increased fatigue (21%), headache (20%), and stomach upset (20%) (Figure 3). 

Although the study was not designed or powered to evaluate differences between treatments, there were several notable differences between the corticosteroid and ACTH groups in the incidence of adverse events, including sleep disturbance (49% versus 15%), increased appetite (29% versus 8%), weight gain (32% versus 8%), and headache (23% versus 0%). Post-hoc analyses indicated a statistically significant difference between groups in sleep disturbance (*P* = 0.035, Fisher exact test).

### 3.4. Total Composite Score, ADL, and RSH

Mean scores for TCS, ADL, and RSH are summarized in Table 5. ADL and RSH scores in Part 1 of the questionnaire were not significantly correlated (Pearson's correlation coefficient = 0.205; *P* = 0.053), but scores in Part 2 were significantly correlated (Pearson's correlation coefficient = 0.723, *P* < 0.0001), suggesting that improvement in ADL is closely related to patients' perception of having returned to their prerelapse baseline.

**Figure 4:**
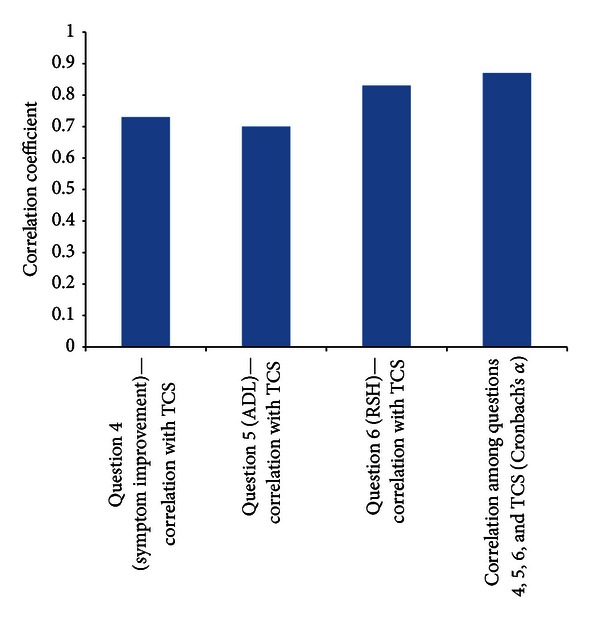
Correlations among total composite score and individual items (Total composite score calculated as the sum of scores from Questions 4, 5, and 6 from Part 2 of the ARMS questionnaire). ADL; activities of daily living; RSH: return to previous state of health; TCS: total composite score.

Analysis of the combined relationship of questions 4 (symptom improvement), 5 (ADL), and 6 (RSH) from Part 2 of the questionnaire with the TCS showed a high Cronbach *α* (0.87), suggesting good internal consistency among those 3 questions. The individual Cronbach *α*'s for Question  4, ADL, and RSH with TCS were 0.84, 0.86, and 0.75, respectively. The corresponding correlation coefficients are shown in Figure 4. There were also significant correlations between Question  4 and ADL (*r* = 0.60, *P* < 0.0001) and RSH (*r* = 0.76, *P* < 0.0001); and as previously noted, ADL was also significantly correlated with RSH (*r* = 0.72, *P* < 0.0001).

## 4. Discussion

The ARMS questionnaire was developed by a working group of MS nurse experts from the United States and Canada, with the objective that it would be employed for the assessment of relapses and evaluation of response to relapse treatment. Results from this pilot study indicate that the ARMS questionnaire appears to be useful for evaluating relapses and response to acute relapse treatment. Cronbach's *α* indicated high internal reliability and consistency among the three interrelated questions in Part 2 (i.e., questions 4 (symptom improvement), 5 (ADL), and 6 (RSH)) and the TCS. High Pearson linear correlations indicated good internal consistency between the ADL and RSH questions in Part 2. The lower correlation between the ADL and RSH questions in Part 1 is not unexpected, as the ADL question refers to the effect of the current relapse on ADL, while the RSH question refers to the effect of treatment for the previous relapse. 

Results obtained with Part 1 of the ARMS questionnaire indicated substantial impact of relapses on patients with new appearance or increased severity of a large number of symptoms, including numbness/tingling, fatigue, leg/foot weakness, and difficulties in walking. Relapses also negatively affected ADL very much or severely in >50% of patients. This finding is consistent with prior results from a very large sample of MS patients which showed that those who experienced one or more relapses in the past 12 months had significantly greater functional disability than patients without relapses over this period [4]. Part 2 results indicated that both corticosteroids and ACTH were effective for treatment of relapses. Approximately 80% of patients reported that treatment improved symptoms and resulted in a return to baseline at least somewhat, and approximately half of patients reported little to no effect of relapse symptoms on ADL following treatment. Although the study was not designed to compare efficacy outcomes between treatments, post-hoc analyses did not identify any statistically significant between-group differences. These results are consistent with results from controlled clinical trials and current treatment guidelines [6, 12, 13]. However, the findings also suggest that there may be some patients whose relapses are not adequately treated with corticosteroids, consistent with the previously mentioned NARCOMS data [9].

Results from Parts 1 and 2 of the ARMS questionnaire indicated that corticosteroid treatment was associated with adverse events, including sleep disturbance, mood changes, and weight gain; all of these are well-known side effects of these agents [14]. A small number of patients included in this study were treated with ACTH; there were considerably lower incidences (differences approximately 20%–30%) of some adverse events (sleep disturbance, increased appetite, weight gain, and headache) among those patients versus patients treated with corticosteroids (although only sleep disturbance was significantly different) and slightly higher incidences (differences ≤6%) of dizziness, fever, high blood sugar, and low blood pressure with ACTH, which were not significantly different between groups. These results contrast with an expert opinion from the National Multiple Sclerosis Society suggesting that the use of ACTH for the treatment of MS relapses may be more likely to result in adverse events than IV corticosteroids [15]. Review of treatments for MS relapses by the European Federation of Neuroscience Societies indicated no consistent differences in the efficacy or safety of IV corticosteroids and ACTH, but this group made no recommendation regarding the use of the latter agents [13]. It should be noted that the AEs reported here were based on the specific list of targeted AEs included in the ARMS questionnaire (as well as additional AEs reported in the open-ended “other” response field), rather than an exhaustive list of any AE, and that the events reported in Part 1 of the questionnaire relied on patient recall. In addition, although these AEs represent those likely to be associated with typical relapse treatments, the nature of the study limits the ability to determine the extent to which the AEs were a result of the specific relapse treatment. Nonetheless, the present results raise the possibility that there may be differences in the mechanisms underlying the physiological effects of these drugs and that further exploration of their relative efficacy and safety profiles may be worthwhile.

The ARMS questionnaire may complement other instruments that have been employed to evaluate the status of patients with MS. The Expanded Disability Status Scale (EDSS) [7] and Multiple Sclerosis Functional Composite (MSFC) [8] are used to delineate the status of patients, and disease progression in MS; and the MSFC has been shown to be sensitive to the occurrence of relapses [16]. There are a large number of self-assessments that have been employed in patients with MS, but they too are not focused on relapses. However, results from one recent study indicated that both the physical and mental component scores and several individual scales of the Medical Outcomes Study Short Form 36, the Modified Fatigue Impact Scale, and the Centers for Epidemiologic Studies Depression Scale are all sensitive to relapses in MS patients [17]. While multiple instruments may be sensitive to MS relapses, it is important to emphasize that none are specifically designed to capture acute changes in relapse-associated symptoms, impact on ADL, and benefits and side effects of treatment. The ARMS questionnaire therefore could be used in conjunction with other assessments to provide clinicians with a more complete picture of how relapses affect their patients. In addition, the internal consistency and reliability among the questions evaluating symptoms, ADL, and RSH following treatment may render the ARMS questionnaire useful for evaluating these outcomes in clinical trial settings; however, further studies would be needed to formally validate the questionnaire for that purpose.

Some aspects of the study design may be considered limitations. For example, the study did not employ strict inclusion or exclusion criteria, and the diagnosis of relapse was at the discretion of the investigator. However, the ARMS questionnaire is intended to be used in a broad range of patients and the study was designed to evaluate the questionnaire's ability to gauge the impact of relapses on patients and its ease of use to clinicians; as such, strict criteria to characterize relapses would limit the generalizability of the findings. In addition, we did not collect other clinical information that may be useful in evaluating differences in outcomes between patients (e.g., age at onset of disease, duration of disease, or use of disease modifying therapies). Finally, the study was not designed to compare outcomes with corticosteroids and ACTH, and the results should be considered with this in mind.

In summary, the results of this pilot study suggest that the ARMS questionnaire can be used in clinical practice settings to evaluate relapses and response to relapse treatments. The ARMS questionnaire may help clinicians to accurately and conveniently assess the nature and impact of relapses as well as the effectiveness of their current approaches to treatment. Furthermore, it may assist in promoting patient-clinician dialogue about relapses and their management across a variety of practice settings. 

## Supplementary Material

The Assessing Relapse in Multiple Sclerosis (ARMS) questionnaire is a 2-part self-report assessment tool developed by a panel of expert MS nurses and specifically designed for evaluating relapses and responses to treatment.

## Figures and Tables

**Table 1:** Demographic and clinical characteristics of patients.

	Patients completing Part 1 of ARMS questionnaire (*N* = 103)
Age, years, mean (SD)	42.5 (11.2)
Sex, *n* (%)	
Male	14 (14)
Female	89 (86)
Type of MS, *n* (%)	(*n* = 97*)
RRMS	95 (98)
SPMS	2 (2)

ARMS: Assessing Relapse in Multiple Sclerosis; SD: standard deviation; RRMS: relapsing remitting MS; SPMS: secondary progressive MS.

*Type of MS was not specified for 6 patients.

**Table 2:** Characteristics of current relapse.

	Patients completing Part 1 of ARMS questionnaire (*N* = 103)
Time since symptoms began, *n* (%)	
≤3 days	8 (8)
4–7 days	26 (25)
8–15 days	30 (29)
≥16 days	39 (38)
Effect of symptoms on ADL, *n* (%)	
A little	9 (9)
Somewhat	35 (34)
Very much	48 (47)
Severely	11 (11)
Mean (SD) ADL score	6.62 (2.1)

ARMS: Assessing Relapse in Multiple Sclerosis; ADL: activities of daily living; SD: standard deviation.

**Table 3:** Outcome for last relapse.

	Patients completing Part 1 of ARMS questionnaire (*N* = 103)
Time since last relapse, months	
Mean (SD)	13.4 (12.0)
Range	0–36
Treatment for last relapse, *n* (%)	
Corticosteroids (IV or oral)	84 (82)
ACTH	5 (5)
Other, no treatment, or not sure	14 (14)
Effect of treatment on RSH, *n* (%)	(*n* = 90*)
No improvement	1 (1)
A little	8 (9)
Somewhat	17 (19)
Very much	41 (46)
Returned to baseline	23 (26)
Mean (SD) RSH score	7.22 (2.5)

ACTH: adrenocorticotropic hormone; ADL: activities of daily living; ARMS: Assessing Relapse in Multiple Sclerosis; IV: intravenous; RSH: return to previous state of health; SD: standard deviation.

*Responses were not provided by 13 patients who reported no treatment or not sure of treatment for their last relapse.

**Table 4:** ARMS Questionnaire Part 2—Treatment and outcomes for current relapse.

	Patients completing Part 2 of ARMS Questionnaire (*N* = 103)			
Treatment for current relapse, *n* (%)				
Any corticosteroids (IV or oral)		90 (87)		
IV corticosteroids		89 (86)		
Oral corticosteroids		1 (1)		
Oral corticosteroids after IV		23 (22)		
ACTH		13 (13)		

	Total (*N* = 103)	Corticosteroids (*n* = 90)	ACTH (*n* = 13)	*P* value*

Completed prescribed treatment, *n* (%)	100 (97)	87 (97)	13 (100)	
Time since treatment completed, days				
Mean (SD)	28.2 (9.7)	29.0 (9.8)	22.2 (5.8)	
Range	8–90	8–90	14–30	
Treatment improved relapse symptoms, *n* (%)				0.756
Got worse	3 (3)	3 (3)	0	
No improvement	6 (6)	5 (6)	1 (8)	
A little	12 (12)	9 (10)	3 (23)	
Somewhat	32 (31)	28 (31)	4 (31)	
Very much	34 (33)	31 (34)	3 (23)	
Completely resolved	16 (16)	14 (16)	2 (15)	
Effect of symptoms on ADL after treatment, *n* (%)				0.228
Not at all	15 (15)	13 (14)	2 (15)	
A little	35 (34)	34 (38)	1 (8)	
Somewhat	37 (36)	30 (33)	7 (54)	
Very much	14 (14)	11 (12)	3 (23)	
Severely	2 (2)	2 (2)	0	
Effect of treatment on RSH, *n* (%)				0.444
Got worse	3 (3)	3 (3)	0	
No improvement	7 (7)	5 (6)	2 (15)	
A little	13 (13)	11 (12)	2 (15)	
Somewhat	35 (34)	29 (32)	6 (46)	
Very much	27 (26)	26 (29)	1 (8)	
Returned to baseline	18 (17)	16 (18)	2 (15)	

*P values based on chi-square test comparing corticosteroids and ACTH treatment groups.

ACTH: adrenocorticotropic hormone; ADL: activities of daily living; ARMS: Assessing Relapse in Multiple Sclerosis; IV: intravenous; RSH: return to previous state of health; SD: standard deviation.

**Table 5:** Total Composite Score, Activities of Daily Living Score, and Return to Previous State of Health Score.

	Patients Completing Part 2 of ARMS Questionnaire			*P* value*
	Total (*N* = 103)	Corticosteroids (*n* = 90)	ACTH (*n* = 13)	
TCS after relapse, mean (SD) (*N* = 100^†^)	18.51 (7.6)	18.92 (7.4)	15.77 (8.8)	0.166
ADL score, mean (SD)				
Part 1—New relapse (*N* = 103)	3.38 (2.1)	3.43 (2.1)	3.00 (1.9)	0.483
Part 2—After relapse treatment (*N* = 103)	6.21 (2.7)	6.34 (2.7)	5.31 (2.8)	0.198
Change (*N* = 103)	2.83 (2.8)	2.91 (2.9)	2.31 (2.4)	0.478
RSH score, mean (SD)				
Part 1—New relapse (*N* = 90^†^)	7.22 (2.5)	7.33 (2.3)	6.50 (3.3)	0.283
Part 2—After relapse treatment (*N* = 100^†^)	6.00 (3.0)	6.15 (3.0)	5.00 (3.2)	0.201
Change (*N* = 87^†^)	−1.11 (2.6)	−1.03 (2.7)	−1.67 (2.2)	0.438
PCS, mean (SD)				
Part 1—New relapse (*N* = 90^†^)	10.52 (3.6)	10.69 (3.4)	9.42 (4.6)	0.251
Part 2—After relapse treatment (*N* = 100^†^)	12.38 (5.2)	12.69 (5.0)	10.31 (6.0)	0.123
Change (*N* = 87^†^)	2.07 (4.4)	2.29 (4.5)	0.67 (3.4)	0.233

*P values based on *t* test comparing corticosteroids and ACTH treatment groups.

^†^ *n* values for RSH and PCS are lower due to patients who did not respond to these questions in Part 1 (*n* = 13 patients who indicated no treatment or not sure of treatment for last relapse) or Part 2 (*n* = 3 patients in corticosteroids group who responded “got worse”, which was not assigned a numerical value).

ACTH: adrenocorticotropic hormone; ADL: activities of daily living; ARMS: Assessing Relapse in Multiple Sclerosis; PCS: partial composite score; RSH: return to previous state of health; SD: standard deviation; TCS: total composite score.
