# Supplementary material for: Assessing Relapse in Multiple Sclerosis Questionnaire: Results of a Pilot Study
Source: Mult Scler Int. 2013 May 26;2013:470476. doi: 10.1155/2013/470476 (PMC3677606; doi:10.1155/2013/470476)
Supplement: Supplementary file 1 — The Assessing Relapse in Multiple Sclerosis (ARMS) questionnaire is a 2-part self-report assessment tool developed by a panel of expert MS nurses and specifically designed for evaluating relapses and responses to treatment. [file 470476.f1.pdf]

MS Relapse Evaluation—New Relapse

Patient's age in years: ______

Patient's sex (circle one): Male Female

---

1) What are the new or worsening symptoms that you are currently experiencing? *(Check all that apply)*

- ☐ Vision changes
- ☐ Chewing/swallowing
- ☐ Hand/arm weakness
- ☐ Bowel problems
- ☐ Fatigue
- ☐ Difficulty walking
- ☐ Other: ___________________
- ☐ Speech changes
- ☐ Numbness/tingling
- ☐ Leg/foot weakness
- ☐ Sexual problems
- ☐ Muscle tightness or stiffness
- ☐ Coordination (tripping, dropping things)
- ☐ Dizziness/poor balance
- ☐ Pain, burning, itching
- ☐ Bladder problems
- ☐ Memory problems
- ☐ Thinking problems

2) When did these symptoms begin? *(Check one)*

☐ Within the last 3 days ☐ 4 -7 days ago ☐ 8 -15 days ago ☐ 16+ days ago

3) How much have these symptoms affected your daily activities or overall function? *(Mark one)*

Not at all	A Little			Somewhat			Very Much			Severely
☐	☐	☐	☐	☐	☐	☐	☐	☐	☐	☐
0	1	2	3	4	5	6	7	8	9	10

4) How many days/months ago was your last relapse (attack, exacerbation) prior to this current episode? _______

5) What treatment did you receive for your last relapse (attack, exacerbation)? *(Check all that apply)*

- ☐ IV steroid infusion
- ☐ Acthar/ACTH injections
- ☐ Other:
- ☐ Oral steroid tablets (only)
- ☐ No treatment *(skip questions 6 and 7)*
- ☐ Oral steroid tablets (after IV steroids)
- ☐ Not sure

6) After treatment for your last relapse (attack, exacerbation), how much did you return to your baseline state of health without any residual relapse symptoms? *(Mark one)*

Got worse	No improvement	A Little			Somewhat			Very Much			Completely returned to baseline
☐	☐	☐	☐	☐	☐	☐	☐	☐	☐	☐	☐
	0	1	2	3	4	5	6	7	8	9	10

7) Have you had any side effects from treatments for previous MS relapses (attacks, exacerbations)? *(Check all that apply)*

- ☐ Mood changes/depression/anxiety
- ☐ Sleep disturbance
- ☐ Stomach upset or heartburn
- ☐ High blood sugar
- ☐ Increased appetite
- ☐ Chills
- ☐ Weight gain
- ☐ Increased blood pressure
- ☐ Headache
- ☐ Increased fatigue
- ☐ Fever
- ☐ Infection
- ☐ Nausea and/or vomiting
- ☐ Low blood pressure
- ☐ Faintness (light headedness)
- ☐ Dizziness
- ☐ Muscle cramps
- ☐ Other:

If you have any questions, please ask your Nurse

For office use only

Date: ______________ Patient initials: ______ ______ ______

Type of MS (circle one): RRMS PPMS SPMS

Type of visit (circle one): Phone Office

Questionnaire completed by (circle one): Patient Office Staff
