# Supplementary material for: Assessing Relapse in Multiple Sclerosis Questionnaire: Results of a Pilot Study
Source: Mult Scler Int. 2013 May 26;2013:470476. doi: 10.1155/2013/470476 (PMC3677606; doi:10.1155/2013/470476)
Supplement: Supplementary file 2 [file 470476.f2.pdf]

MS Relapse Evaluation—After Relapse Treatment *(1 month [± 1 week] follow up)*

Patient's age in years: ______

Patient's sex (circle one): Male Female

---

1) What treatment was prescribed for this most recent relapse (attack, exacerbation)? *(Check all that apply)*

☐ IV steroid infusion ☐ Oral steroid tablets (only) ☐ Oral steroid tablets (after IV steroids)
☐ Acthar/ACTH injections ☐ Plasma exchange ☐ No treatment
☐ I'm not sure ☐ Other: _______________

2) Did you complete the prescribed relapse treatment?

☐ Yes ☐ No If not, why? ____

3) How many days/months has it been since you completed treatment for this most recent relapse (attack, exacerbation)? _____

4) Do you think that the treatment for this most recent relapse (attack, exacerbation) resulted in improvement of your relapse symptoms? *(Mark one)*

Got worse	No improvement	A Little			Somewhat			Very Much			Completely resolved
☐	☐	☐	☐	☐	☐	☐	☐	☐	☐	☐	☐
	0	1	2	3	4	5	6	7	8	9	10

5) Following treatment for this most recent relapse (attack, exacerbation), how much are your relapse symptoms continuing to affect your daily activities or overall function? *(Mark one)*

Not at all	A Little			Somewhat			Very Much			Severely
☐	☐	☐	☐	☐	☐	☐	☐	☐	☐	☐
0	1	2	3	4	5	6	7	8	9	10

6) Following treatment for this most recent relapse (attack, exacerbation), how much do you feel that you have returned to your baseline state of health without any residual relapse symptoms? *(Mark one)*

Got worse	No improvement	A Little			Somewhat			Very Much			Completely returned to baseline
☐	☐	☐	☐	☐	☐	☐	☐	☐	☐	☐	☐
	0	1	2	3	4	5	6	7	8	9	10

7) Did you have any side effects from the treatment you received for this relapse (attack, exacerbation)? *(Check all that apply)*

☐ Mood changes/depression/anxiety ☐ Weight gain ☐ Nausea and/or vomiting
☐ Sleep disturbance ☐ Increased blood pressure ☐ Low blood pressure
☐ Stomach upset or heartburn ☐ Headache ☐ Faintness (light headedness)
☐ High blood sugar ☐ Increased fatigue ☐ Dizziness
☐ Increased appetite ☐ Fever ☐ Muscle cramps
☐ Chills ☐ Infection ☐ Other:

If you have any questions, please ask your MS Nurse

For office use only

Date: _____________ Patient initials: ______ ______ ______

Type of MS (circle one): RRMS PPMS SPMS

Type of visit (circle one): Phone Office

Questionnaire completed by (circle one): Patient Office Staff

MS Relapse Evaluation—After Relapse Treatment *(1 month [± 1 week] follow up)*

Patient's age in years: ______

Patient's sex (circle one): Male Female

---

1) What treatment was prescribed for this most recent relapse (attack, exacerbation)? *(Check all that apply)*

- ☐ IV steroid infusion
- ☐ Oral steroid tablets (only)
- ☐ Oral steroid tablets (after IV steroids)
- ☐ Acthar/ACTH injections
- ☐ Plasma exchange
- ☐ No treatment
- ☐ I'm not sure
- ☐ Other: _______________

2) Did you complete the prescribed relapse treatment?

☐ Yes ☐ No If not, why? ____

3) How many days/months has it been since you completed treatment for this most recent relapse (attack, exacerbation)? _____

4) Do you think that the treatment for this most recent relapse (attack, exacerbation) resulted in improvement of your relapse symptoms? *(Mark one)*

Got worse	No improvement	A Little			Somewhat			Very Much			Completely resolved
☐	☐	☐	☐	☐	☐	☐	☐	☐	☐	☐	☐
	0	1	2	3	4	5	6	7	8	9	10

5) Following treatment for this most recent relapse (attack, exacerbation), how much are your relapse symptoms continuing to affect your daily activities or overall function? *(Mark one)*

Not at all	A Little			Somewhat			Very Much			Severely
☐	☐	☐	☐	☐	☐	☐	☐	☐	☐	☐
0	1	2	3	4	5	6	7	8	9	10

6) Following treatment for this most recent relapse (attack, exacerbation), how much do you feel that you have returned to your baseline state of health without any residual relapse symptoms? *(Mark one)*

Got worse	No improvement	A Little			Somewhat			Very Much			Completely returned to baseline
☐	☐	☐	☐	☐	☐	☐	☐	☐	☐	☐	☐
	0	1	2	3	4	5	6	7	8	9	10

7) Did you have any side effects from the treatment you received for this relapse (attack, exacerbation)? *(Check all that apply)*

- ☐ Mood changes/depression/anxiety
- ☐ Sleep disturbance
- ☐ Stomach upset or heartburn
- ☐ High blood sugar
- ☐ Increased appetite
- ☐ Chills
- ☐ Weight gain
- ☐ Increased blood pressure
- ☐ Headache
- ☐ Increased fatigue
- ☐ Fever
- ☐ Infection
- ☐ Nausea and/or vomiting
- ☐ Low blood pressure
- ☐ Faintness (light headedness)
- ☐ Dizziness
- ☐ Muscle cramps
- ☐ Other:

If you have any questions, please ask your MS Nurse

For office use only

Date: _____________ Patient initials: ______ ______ ______

Type of MS (circle one): RRMS PPMS SPMS

Type of visit (circle one): Phone Office

Questionnaire completed by (circle one): Patient Office Staff

MS Relapse Evaluation—After Relapse Treatment *(1 month [± 1 week] follow up)*

Patient's age in years: ______

Patient's sex (circle one): Male Female

---

1) What treatment was prescribed for this most recent relapse (attack, exacerbation)? *(Check all that apply)*

- ☐ IV steroid infusion
- ☐ Oral steroid tablets (only)
- ☐ Oral steroid tablets (after IV steroids)
- ☐ Acthar/ACTH injections
- ☐ Plasma exchange
- ☐ No treatment
- ☐ I'm not sure
- ☐ Other: _______________

2) Did you complete the prescribed relapse treatment?

☐ Yes ☐ No If not, why? ____

3) How many days/months has it been since you completed treatment for this most recent relapse (attack, exacerbation)? _____

4) Do you think that the treatment for this most recent relapse (attack, exacerbation) resulted in improvement of your relapse symptoms? *(Mark one)*

Got worse	No improvement	A Little			Somewhat			Very Much			Completely resolved
☐	☐	☐	☐	☐	☐	☐	☐	☐	☐	☐	☐
	0	1	2	3	4	5	6	7	8	9	10

5) Following treatment for this most recent relapse (attack, exacerbation), how much are your relapse symptoms continuing to affect your daily activities or overall function? *(Mark one)*

Not at all	A Little			Somewhat			Very Much			Severely
☐	☐	☐	☐	☐	☐	☐	☐	☐	☐	☐
0	1	2	3	4	5	6	7	8	9	10

6) Following treatment for this most recent relapse (attack, exacerbation), how much do you feel that you have returned to your baseline state of health without any residual relapse symptoms? *(Mark one)*

Got worse	No improvement	A Little			Somewhat			Very Much			Completely returned to baseline
☐	☐	☐	☐	☐	☐	☐	☐	☐	☐	☐	☐
	0	1	2	3	4	5	6	7	8	9	10

7) Did you have any side effects from the treatment you received for this relapse (attack, exacerbation)? *(Check all that apply)*

- ☐ Mood changes/depression/anxiety
- ☐ Weight gain
- ☐ Nausea and/or vomiting
- ☐ Sleep disturbance
- ☐ Increased blood pressure
- ☐ Low blood pressure
- ☐ Stomach upset or heartburn
- ☐ Headache
- ☐ Faintness (light headedness)
- ☐ High blood sugar
- ☐ Increased fatigue
- ☐ Dizziness
- ☐ Increased appetite
- ☐ Fever
- ☐ Muscle cramps
- ☐ Chills
- ☐ Infection
- ☐ Other:

If you have any questions, please ask your MS Nurse

For office use only

Date: _____________ Patient initials: ______ ______ ______

Type of MS (circle one): RRMS PPMS SPMS

Type of visit (circle one): Phone Office

Questionnaire completed by (circle one): Patient Office Staff

MS Relapse Evaluation—After Relapse Treatment *(1 month [± 1 week] follow up)*

Patient's age in years: ______

Patient's sex (circle one): Male Female

---

1) What treatment was prescribed for this most recent relapse (attack, exacerbation)? *(Check all that apply)*

☐ IV steroid infusion ☐ Oral steroid tablets (only) ☐ Oral steroid tablets (after IV steroids)
☐ Acthar/ACTH injections ☐ Plasma exchange ☐ No treatment
☐ I'm not sure ☐ Other: _______________

2) Did you complete the prescribed relapse treatment?

☐ Yes ☐ No If not, why? ______________________________

3) How many days/months has it been since you completed treatment for this most recent relapse (attack, exacerbation)? _____

4) Do you think that the treatment for this most recent relapse (attack, exacerbation) resulted in improvement of your relapse symptoms? *(Mark one)*

Got worse	No improvement	A Little			Somewhat			Very Much			Completely resolved
☐	☐	☐	☐	☐	☐	☐	☐	☐	☐	☐	☐
	0	1	2	3	4	5	6	7	8	9	10

5) Following treatment for this most recent relapse (attack, exacerbation), how much are your relapse symptoms continuing to affect your daily activities or overall function? *(Mark one)*

Not at all	A Little			Somewhat			Very Much			Severely
☐	☐	☐	☐	☐	☐	☐	☐	☐	☐	☐
0	1	2	3	4	5	6	7	8	9	10

6) Following treatment for this most recent relapse (attack, exacerbation), how much do you feel that you have returned to your baseline state of health without any residual relapse symptoms? *(Mark one)*

Got worse	No improvement	A Little			Somewhat			Very Much			Completely returned to baseline
☐	☐	☐	☐	☐	☐	☐	☐	☐	☐	☐	☐
	0	1	2	3	4	5	6	7	8	9	10

7) Did you have any side effects from the treatment you received for this relapse (attack, exacerbation)? *(Check all that apply)*

☐ Mood changes/depression/anxiety ☐ Weight gain ☐ Nausea and/or vomiting
☐ Sleep disturbance ☐ Increased blood pressure ☐ Low blood pressure
☐ Stomach upset or heartburn ☐ Headache ☐ Faintness (light headedness)
☐ High blood sugar ☐ Increased fatigue ☐ Dizziness
☐ Increased appetite ☐ Fever ☐ Muscle cramps
☐ Chills ☐ Infection ☐ Other:

If you have any questions, please ask your MS Nurse

For office use only

Date: _____________ Patient initials: ______ ______ ______

Type of MS (circle one): RRMS PPMS SPMS

Type of visit (circle one): Phone Office

Questionnaire completed by (circle one): Patient Office Staff

MS Relapse Evaluation—After Relapse Treatment *(1 month [± 1 week] follow up)*

Patient's age in years: ______

Patient's sex (circle one): Male Female

---

1) What treatment was prescribed for this most recent relapse (attack, exacerbation)? *(Check all that apply)*

- ☐ IV steroid infusion
- ☐ Oral steroid tablets (only)
- ☐ Oral steroid tablets (after IV steroids)
- ☐ Acthar/ACTH injections
- ☐ Plasma exchange
- ☐ No treatment
- ☐ I'm not sure
- ☐ Other: ______________

2) Did you complete the prescribed relapse treatment?

☐ Yes ☐ No If not, why? ____

3) How many days/months has it been since you completed treatment for this most recent relapse (attack, exacerbation)? _____

4) Do you think that the treatment for this most recent relapse (attack, exacerbation) resulted in improvement of your relapse symptoms? *(Mark one)*

Got worse	No improvement	A Little			Somewhat			Very Much			Completely resolved
☐	☐	☐	☐	☐	☐	☐	☐	☐	☐	☐	☐
	0	1	2	3	4	5	6	7	8	9	10

5) Following treatment for this most recent relapse (attack, exacerbation), how much are your relapse symptoms continuing to affect your daily activities or overall function? *(Mark one)*

Not at all	A Little			Somewhat			Very Much			Severely
☐	☐	☐	☐	☐	☐	☐	☐	☐	☐	☐
0	1	2	3	4	5	6	7	8	9	10

6) Following treatment for this most recent relapse (attack, exacerbation), how much do you feel that you have returned to your baseline state of health without any residual relapse symptoms? *(Mark one)*

Got worse	No improvement	A Little			Somewhat			Very Much			Completely returned to baseline
☐	☐	☐	☐	☐	☐	☐	☐	☐	☐	☐	☐
	0	1	2	3	4	5	6	7	8	9	10

7) Did you have any side effects from the treatment you received for this relapse (attack, exacerbation)? *(Check all that apply)*

- ☐ Mood changes/depression/anxiety
- ☐ Sleep disturbance
- ☐ Stomach upset or heartburn
- ☐ High blood sugar
- ☐ Increased appetite
- ☐ Chills
- ☐ Weight gain
- ☐ Increased blood pressure
- ☐ Headache
- ☐ Increased fatigue
- ☐ Fever
- ☐ Infection
- ☐ Nausea and/or vomiting
- ☐ Low blood pressure
- ☐ Faintness (light headedness)
- ☐ Dizziness
- ☐ Muscle cramps
- ☐ Other:

If you have any questions, please ask your MS Nurse

For office use only

Date: ____________ Patient initials: ______ ______ ______

Type of MS (circle one): RRMS PPMS SPMS

Type of visit (circle one): Phone Office

Questionnaire completed by (circle one): Patient Office Staff

MS Relapse Evaluation—After Relapse Treatment *(1 month [± 1 week] follow up)*

Patient's age in years: ______

Patient's sex (circle one): Male Female

---

1) What treatment was prescribed for this most recent relapse (attack, exacerbation)? *(Check all that apply)*

- ☐ IV steroid infusion
- ☐ Oral steroid tablets (only)
- ☐ Oral steroid tablets (after IV steroids)
- ☐ Acthar/ACTH injections
- ☐ Plasma exchange
- ☐ No treatment
- ☐ I'm not sure
- ☐ Other: _______________

2) Did you complete the prescribed relapse treatment?

☐ Yes ☐ No If not, why? ______________________________

3) How many days/months has it been since you completed treatment for this most recent relapse (attack, exacerbation)? _____

4) Do you think that the treatment for this most recent relapse (attack, exacerbation) resulted in improvement of your relapse symptoms? *(Mark one)*

Got worse	No improvement	A Little			Somewhat			Very Much			Completely resolved
☐	☐	☐	☐	☐	☐	☐	☐	☐	☐	☐	☐
	0	1	2	3	4	5	6	7	8	9	10

5) Following treatment for this most recent relapse (attack, exacerbation), how much are your relapse symptoms continuing to affect your daily activities or overall function? *(Mark one)*

Not at all	A Little			Somewhat			Very Much			Severely
☐	☐	☐	☐	☐	☐	☐	☐	☐	☐	☐
0	1	2	3	4	5	6	7	8	9	10

6) Following treatment for this most recent relapse (attack, exacerbation), how much do you feel that you have returned to your baseline state of health without any residual relapse symptoms? *(Mark one)*

Got worse	No improvement	A Little			Somewhat			Very Much			Completely returned to baseline
☐	☐	☐	☐	☐	☐	☐	☐	☐	☐	☐	☐
	0	1	2	3	4	5	6	7	8	9	10

7) Did you have any side effects from the treatment you received for this relapse (attack, exacerbation)? *(Check all that apply)*

- ☐ Mood changes/depression/anxiety
- ☐ Weight gain
- ☐ Nausea and/or vomiting
- ☐ Sleep disturbance
- ☐ Increased blood pressure
- ☐ Low blood pressure
- ☐ Stomach upset or heartburn
- ☐ Headache
- ☐ Faintness (light headedness)
- ☐ High blood sugar
- ☐ Increased fatigue
- ☐ Dizziness
- ☐ Increased appetite
- ☐ Fever
- ☐ Muscle cramps
- ☐ Chills
- ☐ Infection
- ☐ Other:

If you have any questions, please ask your MS Nurse

For office use only

Date: _____________ Patient initials: ______ ______ ______

Type of MS (circle one): RRMS PPMS SPMS

Type of visit (circle one): Phone Office

Questionnaire completed by (circle one): Patient Office Staff

MS Relapse Evaluation—After Relapse Treatment *(1 month [± 1 week] follow up)*

Patient's age in years: ______

Patient's sex (circle one): Male Female

---

1) What treatment was prescribed for this most recent relapse (attack, exacerbation)? *(Check all that apply)*

- ☐ IV steroid infusion
- ☐ Oral steroid tablets (only)
- ☐ Oral steroid tablets (after IV steroids)
- ☐ Acthar/ACTH injections
- ☐ Plasma exchange
- ☐ No treatment
- ☐ I'm not sure
- ☐ Other: _______________

2) Did you complete the prescribed relapse treatment?

☐ Yes ☐ No If not, why? ____

3) How many days/months has it been since you completed treatment for this most recent relapse (attack, exacerbation)? _____

4) Do you think that the treatment for this most recent relapse (attack, exacerbation) resulted in improvement of your relapse symptoms? *(Mark one)*

Got worse	No improvement	A Little			Somewhat			Very Much			Completely resolved
☐	☐	☐	☐	☐	☐	☐	☐	☐	☐	☐	☐
	0	1	2	3	4	5	6	7	8	9	10

5) Following treatment for this most recent relapse (attack, exacerbation), how much are your relapse symptoms continuing to affect your daily activities or overall function? *(Mark one)*

Not at all	A Little			Somewhat			Very Much			Severely
☐	☐	☐	☐	☐	☐	☐	☐	☐	☐	☐
0	1	2	3	4	5	6	7	8	9	10

6) Following treatment for this most recent relapse (attack, exacerbation), how much do you feel that you have returned to your baseline state of health without any residual relapse symptoms? *(Mark one)*

Got worse	No improvement	A Little			Somewhat			Very Much			Completely returned to baseline
☐	☐	☐	☐	☐	☐	☐	☐	☐	☐	☐	☐
	0	1	2	3	4	5	6	7	8	9	10

7) Did you have any side effects from the treatment you received for this relapse (attack, exacerbation)? *(Check all that apply)*

- ☐ Mood changes/depression/anxiety
- ☐ Sleep disturbance
- ☐ Stomach upset or heartburn
- ☐ High blood sugar
- ☐ Increased appetite
- ☐ Chills
- ☐ Weight gain
- ☐ Increased blood pressure
- ☐ Headache
- ☐ Increased fatigue
- ☐ Fever
- ☐ Infection
- ☐ Nausea and/or vomiting
- ☐ Low blood pressure
- ☐ Faintness (light headedness)
- ☐ Dizziness
- ☐ Muscle cramps
- ☐ Other:

If you have any questions, please ask your MS Nurse

For office use only

Date: _____________ Patient initials: ______ ______ ______

Type of MS (circle one): RRMS PPMS SPMS

Type of visit (circle one): Phone Office

Questionnaire completed by (circle one): Patient Office Staff

MS Relapse Evaluation—After Relapse Treatment *(1 month [± 1 week] follow up)*

Patient's age in years: ______

Patient's sex (circle one): Male Female

---

1) What treatment was prescribed for this most recent relapse (attack, exacerbation)? *(Check all that apply)*

- ☐ IV steroid infusion
- ☐ Oral steroid tablets (only)
- ☐ Oral steroid tablets (after IV steroids)
- ☐ Acthar/ACTH injections
- ☐ Plasma exchange
- ☐ No treatment
- ☐ I'm not sure
- ☐ Other: ______________

2) Did you complete the prescribed relapse treatment?

☐ Yes ☐ No If not, why? ____

3) How many days/months has it been since you completed treatment for this most recent relapse (attack, exacerbation)? _____

4) Do you think that the treatment for this most recent relapse (attack, exacerbation) resulted in improvement of your relapse symptoms? *(Mark one)*

Got worse	No improvement	A Little			Somewhat			Very Much			Completely resolved
☐	☐	☐	☐	☐	☐	☐	☐	☐	☐	☐	☐
	0	1	2	3	4	5	6	7	8	9	10

5) Following treatment for this most recent relapse (attack, exacerbation), how much are your relapse symptoms continuing to affect your daily activities or overall function? *(Mark one)*

Not at all	A Little			Somewhat			Very Much			Severely
☐	☐	☐	☐	☐	☐	☐	☐	☐	☐	☐
0	1	2	3	4	5	6	7	8	9	10

6) Following treatment for this most recent relapse (attack, exacerbation), how much do you feel that you have returned to your baseline state of health without any residual relapse symptoms? *(Mark one)*

Got worse	No improvement	A Little			Somewhat			Very Much			Completely returned to baseline
☐	☐	☐	☐	☐	☐	☐	☐	☐	☐	☐	☐
	0	1	2	3	4	5	6	7	8	9	10

7) Did you have any side effects from the treatment you received for this relapse (attack, exacerbation)? *(Check all that apply)*

- ☐ Mood changes/depression/anxiety
- ☐ Weight gain
- ☐ Nausea and/or vomiting
- ☐ Sleep disturbance
- ☐ Increased blood pressure
- ☐ Low blood pressure
- ☐ Stomach upset or heartburn
- ☐ Headache
- ☐ Faintness (light headedness)
- ☐ High blood sugar
- ☐ Increased fatigue
- ☐ Dizziness
- ☐ Increased appetite
- ☐ Fever
- ☐ Muscle cramps
- ☐ Chills
- ☐ Infection
- ☐ Other:

If you have any questions, please ask your MS Nurse

For office use only

Date: ____________ Patient initials: ______ ______ ______

Type of MS (circle one): RRMS PPMS SPMS

Type of visit (circle one): Phone Office

Questionnaire completed by (circle one): Patient Office Staff

MS Relapse Evaluation—After Relapse Treatment *(1 month [± 1 week] follow up)*

Patient's age in years: ______

Patient's sex (circle one): Male Female

---

1) What treatment was prescribed for this most recent relapse (attack, exacerbation)? *(Check all that apply)*

- ☐ IV steroid infusion
- ☐ Oral steroid tablets (only)
- ☐ Oral steroid tablets (after IV steroids)
- ☐ Acthar/ACTH injections
- ☐ Plasma exchange
- ☐ No treatment
- ☐ I'm not sure
- ☐ Other: _______________

2) Did you complete the prescribed relapse treatment?

☐ Yes ☐ No If not, why? ____

3) How many days/months has it been since you completed treatment for this most recent relapse (attack, exacerbation)? _____

4) Do you think that the treatment for this most recent relapse (attack, exacerbation) resulted in improvement of your relapse symptoms? *(Mark one)*

Got worse	No improvement	A Little			Somewhat			Very Much			Completely resolved
☐	☐	☐	☐	☐	☐	☐	☐	☐	☐	☐	☐
	0	1	2	3	4	5	6	7	8	9	10

5) Following treatment for this most recent relapse (attack, exacerbation), how much are your relapse symptoms continuing to affect your daily activities or overall function? *(Mark one)*

Not at all	A Little			Somewhat			Very Much			Severely
☐	☐	☐	☐	☐	☐	☐	☐	☐	☐	☐
0	1	2	3	4	5	6	7	8	9	10

6) Following treatment for this most recent relapse (attack, exacerbation), how much do you feel that you have returned to your baseline state of health without any residual relapse symptoms? *(Mark one)*

Got worse	No improvement	A Little			Somewhat			Very Much			Completely returned to baseline
☐	☐	☐	☐	☐	☐	☐	☐	☐	☐	☐	☐
	0	1	2	3	4	5	6	7	8	9	10

7) Did you have any side effects from the treatment you received for this relapse (attack, exacerbation)? *(Check all that apply)*

- ☐ Mood changes/depression/anxiety
- ☐ Sleep disturbance
- ☐ Stomach upset or heartburn
- ☐ High blood sugar
- ☐ Increased appetite
- ☐ Chills
- ☐ Weight gain
- ☐ Increased blood pressure
- ☐ Headache
- ☐ Increased fatigue
- ☐ Fever
- ☐ Infection
- ☐ Nausea and/or vomiting
- ☐ Low blood pressure
- ☐ Faintness (light headedness)
- ☐ Dizziness
- ☐ Muscle cramps
- ☐ Other:

If you have any questions, please ask your MS Nurse

For office use only

Date: _____________ Patient initials: ______ ______ ______

Type of MS (circle one): RRMS PPMS SPMS

Type of visit (circle one): Phone Office

Questionnaire completed by (circle one): Patient Office Staff

MS Relapse Evaluation—After Relapse Treatment *(1 month [± 1 week] follow up)*

Patient's age in years: ______

Patient's sex (circle one): Male Female

---

1) What treatment was prescribed for this most recent relapse (attack, exacerbation)? *(Check all that apply)*

- ☐ IV steroid infusion
- ☐ Oral steroid tablets (only)
- ☐ Oral steroid tablets (after IV steroids)
- ☐ Acthar/ACTH injections
- ☐ Plasma exchange
- ☐ No treatment
- ☐ I'm not sure
- ☐ Other: _______________

2) Did you complete the prescribed relapse treatment?

☐ Yes ☐ No If not, why? ______________________________

3) How many days/months has it been since you completed treatment for this most recent relapse (attack, exacerbation)? _____

4) Do you think that the treatment for this most recent relapse (attack, exacerbation) resulted in improvement of your relapse symptoms? *(Mark one)*

Got worse	No improvement	A Little			Somewhat			Very Much			Completely resolved
☐	☐	☐	☐	☐	☐	☐	☐	☐	☐	☐	☐
	0	1	2	3	4	5	6	7	8	9	10

5) Following treatment for this most recent relapse (attack, exacerbation), how much are your relapse symptoms continuing to affect your daily activities or overall function? *(Mark one)*

Not at all	A Little			Somewhat			Very Much			Severely
☐	☐	☐	☐	☐	☐	☐	☐	☐	☐	☐
0	1	2	3	4	5	6	7	8	9	10

6) Following treatment for this most recent relapse (attack, exacerbation), how much do you feel that you have returned to your baseline state of health without any residual relapse symptoms? *(Mark one)*

Got worse	No improvement	A Little			Somewhat			Very Much			Completely returned to baseline
☐	☐	☐	☐	☐	☐	☐	☐	☐	☐	☐	☐
	0	1	2	3	4	5	6	7	8	9	10

7) Did you have any side effects from the treatment you received for this relapse (attack, exacerbation)? *(Check all that apply)*

- ☐ Mood changes/depression/anxiety
- ☐ Weight gain
- ☐ Nausea and/or vomiting
- ☐ Sleep disturbance
- ☐ Increased blood pressure
- ☐ Low blood pressure
- ☐ Stomach upset or heartburn
- ☐ Headache
- ☐ Faintness (light headedness)
- ☐ High blood sugar
- ☐ Increased fatigue
- ☐ Dizziness
- ☐ Increased appetite
- ☐ Fever
- ☐ Muscle cramps
- ☐ Chills
- ☐ Infection
- ☐ Other:

If you have any questions, please ask your MS Nurse

For office use only

Date: _____________ Patient initials: ______ ______ ______

Type of MS (circle one): RRMS PPMS SPMS

Type of visit (circle one): Phone Office

Questionnaire completed by (circle one): Patient Office Staff

MS Relapse Evaluation—After Relapse Treatment *(1 month [± 1 week] follow up)*

Patient's age in years: ______

Patient's sex (circle one): Male Female

---

1) What treatment was prescribed for this most recent relapse (attack, exacerbation)? *(Check all that apply)*

- ☐ IV steroid infusion
- ☐ Oral steroid tablets (only)
- ☐ Oral steroid tablets (after IV steroids)
- ☐ Acthar/ACTH injections
- ☐ Plasma exchange
- ☐ No treatment
- ☐ I'm not sure
- ☐ Other: ________________

2) Did you complete the prescribed relapse treatment?

☐ Yes ☐ No If not, why? ____

3) How many days/months has it been since you completed treatment for this most recent relapse (attack, exacerbation)? _____

4) Do you think that the treatment for this most recent relapse (attack, exacerbation) resulted in improvement of your relapse symptoms? *(Mark one)*

Got worse	No improvement	A Little			Somewhat			Very Much			Completely resolved
☐	☐	☐	☐	☐	☐	☐	☐	☐	☐	☐	☐
	0	1	2	3	4	5	6	7	8	9	10

5) Following treatment for this most recent relapse (attack, exacerbation), how much are your relapse symptoms continuing to affect your daily activities or overall function? *(Mark one)*

Not at all	A Little			Somewhat			Very Much			Severely
☐	☐	☐	☐	☐	☐	☐	☐	☐	☐	☐
0	1	2	3	4	5	6	7	8	9	10

6) Following treatment for this most recent relapse (attack, exacerbation), how much do you feel that you have returned to your baseline state of health without any residual relapse symptoms? *(Mark one)*

Got worse	No improvement	A Little			Somewhat			Very Much			Completely returned to baseline
☐	☐	☐	☐	☐	☐	☐	☐	☐	☐	☐	☐
	0	1	2	3	4	5	6	7	8	9	10

7) Did you have any side effects from the treatment you received for this relapse (attack, exacerbation)? *(Check all that apply)*

- ☐ Mood changes/depression/anxiety
- ☐ Weight gain
- ☐ Nausea and/or vomiting
- ☐ Sleep disturbance
- ☐ Increased blood pressure
- ☐ Low blood pressure
- ☐ Stomach upset or heartburn
- ☐ Headache
- ☐ Faintness (light headedness)
- ☐ High blood sugar
- ☐ Increased fatigue
- ☐ Dizziness
- ☐ Increased appetite
- ☐ Fever
- ☐ Muscle cramps
- ☐ Chills
- ☐ Infection
- ☐ Other:

If you have any questions, please ask your MS Nurse

For office use only

Date: ______________ Patient initials: ______ ______ ______

Type of MS (circle one): RRMS PPMS SPMS

Type of visit (circle one): Phone Office

Questionnaire completed by (circle one): Patient Office Staff

MS Relapse Evaluation—After Relapse Treatment *(1 month [± 1 week] follow up)*

Patient's age in years: ______

Patient's sex (circle one): Male Female

---

1) What treatment was prescribed for this most recent relapse (attack, exacerbation)? *(Check all that apply)*

- ☐ IV steroid infusion
- ☐ Oral steroid tablets (only)
- ☐ Oral steroid tablets (after IV steroids)
- ☐ Acthar/ACTH injections
- ☐ Plasma exchange
- ☐ No treatment
- ☐ I'm not sure
- ☐ Other: _______________

2) Did you complete the prescribed relapse treatment?

☐ Yes ☐ No If not, why? ____

3) How many days/months has it been since you completed treatment for this most recent relapse (attack, exacerbation)? _____

4) Do you think that the treatment for this most recent relapse (attack, exacerbation) resulted in improvement of your relapse symptoms? *(Mark one)*

Got worse	No improvement	A Little			Somewhat			Very Much			Completely resolved
☐	☐	☐	☐	☐	☐	☐	☐	☐	☐	☐	☐
	0	1	2	3	4	5	6	7	8	9	10

5) Following treatment for this most recent relapse (attack, exacerbation), how much are your relapse symptoms continuing to affect your daily activities or overall function? *(Mark one)*

Not at all	A Little			Somewhat			Very Much			Severely
☐	☐	☐	☐	☐	☐	☐	☐	☐	☐	☐
0	1	2	3	4	5	6	7	8	9	10

6) Following treatment for this most recent relapse (attack, exacerbation), how much do you feel that you have returned to your baseline state of health without any residual relapse symptoms? *(Mark one)*

Got worse	No improvement	A Little			Somewhat			Very Much			Completely returned to baseline
☐	☐	☐	☐	☐	☐	☐	☐	☐	☐	☐	☐
	0	1	2	3	4	5	6	7	8	9	10

7) Did you have any side effects from the treatment you received for this relapse (attack, exacerbation)? *(Check all that apply)*

- ☐ Mood changes/depression/anxiety
- ☐ Weight gain
- ☐ Nausea and/or vomiting
- ☐ Sleep disturbance
- ☐ Increased blood pressure
- ☐ Low blood pressure
- ☐ Stomach upset or heartburn
- ☐ Headache
- ☐ Faintness (light headedness)
- ☐ High blood sugar
- ☐ Increased fatigue
- ☐ Dizziness
- ☐ Increased appetite
- ☐ Fever
- ☐ Muscle cramps
- ☐ Chills
- ☐ Infection
- ☐ Other:

If you have any questions, please ask your MS Nurse

For office use only

Date: _____________ Patient initials: ______ ______ ______

Type of MS (circle one): RRMS PPMS SPMS

Type of visit (circle one): Phone Office

Questionnaire completed by (circle one): Patient Office Staff

MS Relapse Evaluation—After Relapse Treatment *(1 month [± 1 week] follow up)*

Patient's age in years: ______

Patient's sex (circle one): Male Female

---

1) What treatment was prescribed for this most recent relapse (attack, exacerbation)? *(Check all that apply)*

- ☐ IV steroid infusion
- ☐ Oral steroid tablets (only)
- ☐ Oral steroid tablets (after IV steroids)
- ☐ Acthar/ACTH injections
- ☐ Plasma exchange
- ☐ No treatment
- ☐ I'm not sure
- ☐ Other: _______________

2) Did you complete the prescribed relapse treatment?

☐ Yes ☐ No If not, why? ______________________________

3) How many days/months has it been since you completed treatment for this most recent relapse (attack, exacerbation)? _____

4) Do you think that the treatment for this most recent relapse (attack, exacerbation) resulted in improvement of your relapse symptoms? *(Mark one)*

Got worse	No improvement	A Little			Somewhat			Very Much			Completely resolved
☐	☐	☐	☐	☐	☐	☐	☐	☐	☐	☐	☐
	0	1	2	3	4	5	6	7	8	9	10

5) Following treatment for this most recent relapse (attack, exacerbation), how much are your relapse symptoms continuing to affect your daily activities or overall function? *(Mark one)*

Not at all	A Little			Somewhat			Very Much			Severely
☐	☐	☐	☐	☐	☐	☐	☐	☐	☐	☐
0	1	2	3	4	5	6	7	8	9	10

6) Following treatment for this most recent relapse (attack, exacerbation), how much do you feel that you have returned to your baseline state of health without any residual relapse symptoms? *(Mark one)*

Got worse	No improvement	A Little			Somewhat			Very Much			Completely returned to baseline
☐	☐	☐	☐	☐	☐	☐	☐	☐	☐	☐	☐
	0	1	2	3	4	5	6	7	8	9	10

7) Did you have any side effects from the treatment you received for this relapse (attack, exacerbation)? *(Check all that apply)*

- ☐ Mood changes/depression/anxiety
- ☐ Sleep disturbance
- ☐ Stomach upset or heartburn
- ☐ High blood sugar
- ☐ Increased appetite
- ☐ Chills
- ☐ Weight gain
- ☐ Increased blood pressure
- ☐ Headache
- ☐ Increased fatigue
- ☐ Fever
- ☐ Infection
- ☐ Nausea and/or vomiting
- ☐ Low blood pressure
- ☐ Faintness (light headedness)
- ☐ Dizziness
- ☐ Muscle cramps
- ☐ Other:

If you have any questions, please ask your MS Nurse

For office use only

Date: _____________ Patient initials: ______ ______ ______

Type of MS (circle one): RRMS PPMS SPMS

Type of visit (circle one): Phone Office

Questionnaire completed by (circle one): Patient Office Staff

MS Relapse Evaluation—After Relapse Treatment *(1 month [± 1 week] follow up)*

Patient's age in years: ______

Patient's sex (circle one): Male Female

---

1) What treatment was prescribed for this most recent relapse (attack, exacerbation)? *(Check all that apply)*

☐ IV steroid infusion ☐ Oral steroid tablets (only) ☐ Oral steroid tablets (after IV steroids)
☐ Acthar/ACTH injections ☐ Plasma exchange ☐ No treatment
☐ I'm not sure ☐ Other: _______________

2) Did you complete the prescribed relapse treatment?

☐ Yes ☐ No If not, why? ____

3) How many days/months has it been since you completed treatment for this most recent relapse (attack, exacerbation)? _____

4) Do you think that the treatment for this most recent relapse (attack, exacerbation) resulted in improvement of your relapse symptoms? *(Mark one)*

Got worse	No improvement	A Little			Somewhat			Very Much			Completely resolved
☐	☐	☐	☐	☐	☐	☐	☐	☐	☐	☐	☐
	0	1	2	3	4	5	6	7	8	9	10

5) Following treatment for this most recent relapse (attack, exacerbation), how much are your relapse symptoms continuing to affect your daily activities or overall function? *(Mark one)*

Not at all	A Little			Somewhat			Very Much			Severely
☐	☐	☐	☐	☐	☐	☐	☐	☐	☐	☐
0	1	2	3	4	5	6	7	8	9	10

6) Following treatment for this most recent relapse (attack, exacerbation), how much do you feel that you have returned to your baseline state of health without any residual relapse symptoms? *(Mark one)*

Got worse	No improvement	A Little			Somewhat			Very Much			Completely returned to baseline
☐	☐	☐	☐	☐	☐	☐	☐	☐	☐	☐	☐
	0	1	2	3	4	5	6	7	8	9	10

7) Did you have any side effects from the treatment you received for this relapse (attack, exacerbation)? *(Check all that apply)*

☐ Mood changes/depression/anxiety ☐ Weight gain ☐ Nausea and/or vomiting
☐ Sleep disturbance ☐ Increased blood pressure ☐ Low blood pressure
☐ Stomach upset or heartburn ☐ Headache ☐ Faintness (light headedness)
☐ High blood sugar ☐ Increased fatigue ☐ Dizziness
☐ Increased appetite ☐ Fever ☐ Muscle cramps
☐ Chills ☐ Infection ☐ Other:

If you have any questions, please ask your MS Nurse

For office use only

Date: _____________ Patient initials: ______ ______ ______

Type of MS (circle one): RRMS PPMS SPMS

Type of visit (circle one): Phone Office

Questionnaire completed by (circle one): Patient Office Staff

MS Relapse Evaluation—After Relapse Treatment *(1 month [± 1 week] follow up)*

Patient's age in years: ______

Patient's sex (circle one): Male Female

---

1) What treatment was prescribed for this most recent relapse (attack, exacerbation)? *(Check all that apply)*

- ☐ IV steroid infusion
- ☐ Oral steroid tablets (only)
- ☐ Oral steroid tablets (after IV steroids)
- ☐ Acthar/ACTH injections
- ☐ Plasma exchange
- ☐ No treatment
- ☐ I'm not sure
- ☐ Other: _______________

2) Did you complete the prescribed relapse treatment?

☐ Yes ☐ No If not, why? ____

3) How many days/months has it been since you completed treatment for this most recent relapse (attack, exacerbation)? _____

4) Do you think that the treatment for this most recent relapse (attack, exacerbation) resulted in improvement of your relapse symptoms? *(Mark one)*

Got worse	No improvement	A Little			Somewhat			Very Much			Completely resolved
☐	☐	☐	☐	☐	☐	☐	☐	☐	☐	☐	☐
	0	1	2	3	4	5	6	7	8	9	10

5) Following treatment for this most recent relapse (attack, exacerbation), how much are your relapse symptoms continuing to affect your daily activities or overall function? *(Mark one)*

Not at all	A Little			Somewhat			Very Much			Severely
☐	☐	☐	☐	☐	☐	☐	☐	☐	☐	☐
0	1	2	3	4	5	6	7	8	9	10

6) Following treatment for this most recent relapse (attack, exacerbation), how much do you feel that you have returned to your baseline state of health without any residual relapse symptoms? *(Mark one)*

Got worse	No improvement	A Little			Somewhat			Very Much			Completely returned to baseline
☐	☐	☐	☐	☐	☐	☐	☐	☐	☐	☐	☐
	0	1	2	3	4	5	6	7	8	9	10

7) Did you have any side effects from the treatment you received for this relapse (attack, exacerbation)? *(Check all that apply)*

- ☐ Mood changes/depression/anxiety
- ☐ Weight gain
- ☐ Nausea and/or vomiting
- ☐ Sleep disturbance
- ☐ Increased blood pressure
- ☐ Low blood pressure
- ☐ Stomach upset or heartburn
- ☐ Headache
- ☐ Faintness (light headedness)
- ☐ High blood sugar
- ☐ Increased fatigue
- ☐ Dizziness
- ☐ Increased appetite
- ☐ Fever
- ☐ Muscle cramps
- ☐ Chills
- ☐ Infection
- ☐ Other:

If you have any questions, please ask your MS Nurse

For office use only

Date: _____________ Patient initials: ______ ______ ______

Type of MS (circle one): RRMS PPMS SPMS

Type of visit (circle one): Phone Office

Questionnaire completed by (circle one): Patient Office Staff

MS Relapse Evaluation—After Relapse Treatment *(1 month [± 1 week] follow up)*

Patient's age in years: ______

Patient's sex (circle one): Male Female

---

1) What treatment was prescribed for this most recent relapse (attack, exacerbation)? *(Check all that apply)*

- ☐ IV steroid infusion
- ☐ Oral steroid tablets (only)
- ☐ Oral steroid tablets (after IV steroids)
- ☐ Acthar/ACTH injections
- ☐ Plasma exchange
- ☐ No treatment
- ☐ I'm not sure
- ☐ Other: _______________

2) Did you complete the prescribed relapse treatment?

☐ Yes ☐ No If not, why? ____

3) How many days/months has it been since you completed treatment for this most recent relapse (attack, exacerbation)? _____

4) Do you think that the treatment for this most recent relapse (attack, exacerbation) resulted in improvement of your relapse symptoms? *(Mark one)*

Got worse	No improvement	A Little			Somewhat			Very Much			Completely resolved
☐	☐	☐	☐	☐	☐	☐	☐	☐	☐	☐	☐
	0	1	2	3	4	5	6	7	8	9	10

5) Following treatment for this most recent relapse (attack, exacerbation), how much are your relapse symptoms continuing to affect your daily activities or overall function? *(Mark one)*

Not at all	A Little			Somewhat			Very Much			Severely
☐	☐	☐	☐	☐	☐	☐	☐	☐	☐	☐
0	1	2	3	4	5	6	7	8	9	10

6) Following treatment for this most recent relapse (attack, exacerbation), how much do you feel that you have returned to your baseline state of health without any residual relapse symptoms? *(Mark one)*

Got worse	No improvement	A Little			Somewhat			Very Much			Completely returned to baseline
☐	☐	☐	☐	☐	☐	☐	☐	☐	☐	☐	☐
	0	1	2	3	4	5	6	7	8	9	10

7) Did you have any side effects from the treatment you received for this relapse (attack, exacerbation)? *(Check all that apply)*

- ☐ Mood changes/depression/anxiety
- ☐ Weight gain
- ☐ Nausea and/or vomiting
- ☐ Sleep disturbance
- ☐ Increased blood pressure
- ☐ Low blood pressure
- ☐ Stomach upset or heartburn
- ☐ Headache
- ☐ Faintness (light headedness)
- ☐ High blood sugar
- ☐ Increased fatigue
- ☐ Dizziness
- ☐ Increased appetite
- ☐ Fever
- ☐ Muscle cramps
- ☐ Chills
- ☐ Infection
- ☐ Other:

If you have any questions, please ask your MS Nurse

For office use only

Date: _____________ Patient initials: ______ ______ ______

Type of MS (circle one): RRMS PPMS SPMS

Type of visit (circle one): Phone Office

Questionnaire completed by (circle one): Patient Office Staff

MS Relapse Evaluation—After Relapse Treatment *(1 month [± 1 week] follow up)*

Patient's age in years: ______

Patient's sex (circle one): Male Female

---

1) What treatment was prescribed for this most recent relapse (attack, exacerbation)? *(Check all that apply)*

- ☐ IV steroid infusion
- ☐ Oral steroid tablets (only)
- ☐ Oral steroid tablets (after IV steroids)
- ☐ Acthar/ACTH injections
- ☐ Plasma exchange
- ☐ No treatment
- ☐ I'm not sure
- ☐ Other: _______________

2) Did you complete the prescribed relapse treatment?

☐ Yes ☐ No If not, why? ______________________________

3) How many days/months has it been since you completed treatment for this most recent relapse (attack, exacerbation)? _____

4) Do you think that the treatment for this most recent relapse (attack, exacerbation) resulted in improvement of your relapse symptoms? *(Mark one)*

Got worse	No improvement	A Little			Somewhat			Very Much			Completely resolved
☐	☐	☐	☐	☐	☐	☐	☐	☐	☐	☐	☐
	0	1	2	3	4	5	6	7	8	9	10

5) Following treatment for this most recent relapse (attack, exacerbation), how much are your relapse symptoms continuing to affect your daily activities or overall function? *(Mark one)*

Not at all	A Little			Somewhat			Very Much			Severely
☐	☐	☐	☐	☐	☐	☐	☐	☐	☐	☐
0	1	2	3	4	5	6	7	8	9	10

6) Following treatment for this most recent relapse (attack, exacerbation), how much do you feel that you have returned to your baseline state of health without any residual relapse symptoms? *(Mark one)*

Got worse	No improvement	A Little			Somewhat			Very Much			Completely returned to baseline
☐	☐	☐	☐	☐	☐	☐	☐	☐	☐	☐	☐
	0	1	2	3	4	5	6	7	8	9	10

7) Did you have any side effects from the treatment you received for this relapse (attack, exacerbation)? *(Check all that apply)*

- ☐ Mood changes/depression/anxiety
- ☐ Sleep disturbance
- ☐ Stomach upset or heartburn
- ☐ High blood sugar
- ☐ Increased appetite
- ☐ Chills
- ☐ Weight gain
- ☐ Increased blood pressure
- ☐ Headache
- ☐ Increased fatigue
- ☐ Fever
- ☐ Infection
- ☐ Nausea and/or vomiting
- ☐ Low blood pressure
- ☐ Faintness (light headedness)
- ☐ Dizziness
- ☐ Muscle cramps
- ☐ Other:

If you have any questions, please ask your MS Nurse

For office use only

Date: _____________ Patient initials: ______ ______ ______

Type of MS (circle one): RRMS PPMS SPMS

Type of visit (circle one): Phone Office

Questionnaire completed by (circle one): Patient Office Staff

MS Relapse Evaluation—After Relapse Treatment *(1 month [± 1 week] follow up)*

Patient's age in years: ______

Patient's sex (circle one): Male Female

---

1) What treatment was prescribed for this most recent relapse (attack, exacerbation)? *(Check all that apply)*

- ☐ IV steroid infusion
- ☐ Oral steroid tablets (only)
- ☐ Oral steroid tablets (after IV steroids)
- ☐ Acthar/ACTH injections
- ☐ Plasma exchange
- ☐ No treatment
- ☐ I'm not sure
- ☐ Other: _______________

2) Did you complete the prescribed relapse treatment?

☐ Yes ☐ No If not, why? ____

3) How many days/months has it been since you completed treatment for this most recent relapse (attack, exacerbation)? _____

4) Do you think that the treatment for this most recent relapse (attack, exacerbation) resulted in improvement of your relapse symptoms? *(Mark one)*

Got worse	No improvement	A Little			Somewhat			Very Much			Completely resolved
☐	☐	☐	☐	☐	☐	☐	☐	☐	☐	☐	☐
	0	1	2	3	4	5	6	7	8	9	10

5) Following treatment for this most recent relapse (attack, exacerbation), how much are your relapse symptoms continuing to affect your daily activities or overall function? *(Mark one)*

Not at all	A Little			Somewhat			Very Much			Severely
☐	☐	☐	☐	☐	☐	☐	☐	☐	☐	☐
0	1	2	3	4	5	6	7	8	9	10

6) Following treatment for this most recent relapse (attack, exacerbation), how much do you feel that you have returned to your baseline state of health without any residual relapse symptoms? *(Mark one)*

Got worse	No improvement	A Little			Somewhat			Very Much			Completely returned to baseline
☐	☐	☐	☐	☐	☐	☐	☐	☐	☐	☐	☐
	0	1	2	3	4	5	6	7	8	9	10

7) Did you have any side effects from the treatment you received for this relapse (attack, exacerbation)? *(Check all that apply)*

- ☐ Mood changes/depression/anxiety
- ☐ Sleep disturbance
- ☐ Stomach upset or heartburn
- ☐ High blood sugar
- ☐ Increased appetite
- ☐ Chills
- ☐ Weight gain
- ☐ Increased blood pressure
- ☐ Headache
- ☐ Increased fatigue
- ☐ Fever
- ☐ Infection
- ☐ Nausea and/or vomiting
- ☐ Low blood pressure
- ☐ Faintness (light headedness)
- ☐ Dizziness
- ☐ Muscle cramps
- ☐ Other:

If you have any questions, please ask your MS Nurse

For office use only

Date: _____________ Patient initials: ______ ______ ______

Type of MS (circle one): RRMS PPMS SPMS

Type of visit (circle one): Phone Office

Questionnaire completed by (circle one): Patient Office Staff

MS Relapse Evaluation—After Relapse Treatment *(1 month [± 1 week] follow up)*

Patient's age in years: ______

Patient's sex (circle one): Male Female

---

1) What treatment was prescribed for this most recent relapse (attack, exacerbation)? *(Check all that apply)*

- ☐ IV steroid infusion
- ☐ Oral steroid tablets (only)
- ☐ Oral steroid tablets (after IV steroids)
- ☐ Acthar/ACTH injections
- ☐ Plasma exchange
- ☐ No treatment
- ☐ I'm not sure
- ☐ Other: _______________

2) Did you complete the prescribed relapse treatment?

☐ Yes ☐ No If not, why? ______________________________

3) How many days/months has it been since you completed treatment for this most recent relapse (attack, exacerbation)? _____

4) Do you think that the treatment for this most recent relapse (attack, exacerbation) resulted in improvement of your relapse symptoms? *(Mark one)*

Got worse	No improvement	A Little			Somewhat			Very Much			Completely resolved
☐	☐	☐	☐	☐	☐	☐	☐	☐	☐	☐	☐
	0	1	2	3	4	5	6	7	8	9	10

5) Following treatment for this most recent relapse (attack, exacerbation), how much are your relapse symptoms continuing to affect your daily activities or overall function? *(Mark one)*

Not at all	A Little			Somewhat			Very Much			Severely
☐	☐	☐	☐	☐	☐	☐	☐	☐	☐	☐
0	1	2	3	4	5	6	7	8	9	10

6) Following treatment for this most recent relapse (attack, exacerbation), how much do you feel that you have returned to your baseline state of health without any residual relapse symptoms? *(Mark one)*

Got worse	No improvement	A Little			Somewhat			Very Much			Completely returned to baseline
☐	☐	☐	☐	☐	☐	☐	☐	☐	☐	☐	☐
	0	1	2	3	4	5	6	7	8	9	10

7) Did you have any side effects from the treatment you received for this relapse (attack, exacerbation)? *(Check all that apply)*

- ☐ Mood changes/depression/anxiety
- ☐ Weight gain
- ☐ Nausea and/or vomiting
- ☐ Sleep disturbance
- ☐ Increased blood pressure
- ☐ Low blood pressure
- ☐ Stomach upset or heartburn
- ☐ Headache
- ☐ Faintness (light headedness)
- ☐ High blood sugar
- ☐ Increased fatigue
- ☐ Dizziness
- ☐ Increased appetite
- ☐ Fever
- ☐ Muscle cramps
- ☐ Chills
- ☐ Infection
- ☐ Other:

If you have any questions, please ask your MS Nurse

For office use only

Date: _____________ Patient initials: ______ ______ ______

Type of MS (circle one): RRMS PPMS SPMS

Type of visit (circle one): Phone Office

Questionnaire completed by (circle one): Patient Office Staff

MS Relapse Evaluation—After Relapse Treatment *(1 month [± 1 week] follow up)*

Patient's age in years: ______

Patient's sex (circle one): Male Female

---

1) What treatment was prescribed for this most recent relapse (attack, exacerbation)? *(Check all that apply)*

☐ IV steroid infusion ☐ Oral steroid tablets (only) ☐ Oral steroid tablets (after IV steroids)
☐ Acthar/ACTH injections ☐ Plasma exchange ☐ No treatment
☐ I'm not sure ☐ Other: _______________

2) Did you complete the prescribed relapse treatment?
☐ Yes ☐ No If not, why? ____

3) How many days/months has it been since you completed treatment for this most recent relapse (attack, exacerbation)? _____

4) Do you think that the treatment for this most recent relapse (attack, exacerbation) resulted in improvement of your relapse symptoms? *(Mark one)*

Got worse	No improvement	A Little			Somewhat			Very Much			Completely resolved
☐	☐	☐	☐	☐	☐	☐	☐	☐	☐	☐	☐
	0	1	2	3	4	5	6	7	8	9	10

5) Following treatment for this most recent relapse (attack, exacerbation), how much are your relapse symptoms continuing to affect your daily activities or overall function? *(Mark one)*

Not at all	A Little			Somewhat			Very Much			Severely
☐	☐	☐	☐	☐	☐	☐	☐	☐	☐	☐
0	1	2	3	4	5	6	7	8	9	10

6) Following treatment for this most recent relapse (attack, exacerbation), how much do you feel that you have returned to your baseline state of health without any residual relapse symptoms? *(Mark one)*

Got worse	No improvement	A Little			Somewhat			Very Much			Completely returned to baseline
☐	☐	☐	☐	☐	☐	☐	☐	☐	☐	☐	☐
	0	1	2	3	4	5	6	7	8	9	10

7) Did you have any side effects from the treatment you received for this relapse (attack, exacerbation)? *(Check all that apply)*

☐ Mood changes/depression/anxiety ☐ Weight gain ☐ Nausea and/or vomiting
☐ Sleep disturbance ☐ Increased blood pressure ☐ Low blood pressure
☐ Stomach upset or heartburn ☐ Headache ☐ Faintness (light headedness)
☐ High blood sugar ☐ Increased fatigue ☐ Dizziness
☐ Increased appetite ☐ Fever ☐ Muscle cramps
☐ Chills ☐ Infection ☐ Other:

If you have any questions, please ask your MS Nurse

For office use only

Date: _____________ Patient initials: ______ ______ ______

Type of MS (circle one): RRMS PPMS SPMS

Type of visit (circle one): Phone Office

Questionnaire completed by (circle one): Patient Office Staff

MS Relapse Evaluation—After Relapse Treatment *(1 month [± 1 week] follow up)*

Patient's age in years: ______

Patient's sex (circle one): Male Female

---

1) What treatment was prescribed for this most recent relapse (attack, exacerbation)? *(Check all that apply)*

- ☐ IV steroid infusion
- ☐ Oral steroid tablets (only)
- ☐ Oral steroid tablets (after IV steroids)
- ☐ Acthar/ACTH injections
- ☐ Plasma exchange
- ☐ No treatment
- ☐ I'm not sure
- ☐ Other: _______________

2) Did you complete the prescribed relapse treatment?

☐ Yes ☐ No If not, why? ______________________________

3) How many days/months has it been since you completed treatment for this most recent relapse (attack, exacerbation)? _____

4) Do you think that the treatment for this most recent relapse (attack, exacerbation) resulted in improvement of your relapse symptoms? *(Mark one)*

Got worse	No improvement	A Little			Somewhat			Very Much			Completely resolved
☐	☐	☐	☐	☐	☐	☐	☐	☐	☐	☐	☐
	0	1	2	3	4	5	6	7	8	9	10

5) Following treatment for this most recent relapse (attack, exacerbation), how much are your relapse symptoms continuing to affect your daily activities or overall function? *(Mark one)*

Not at all	A Little			Somewhat			Very Much			Severely
☐	☐	☐	☐	☐	☐	☐	☐	☐	☐	☐
0	1	2	3	4	5	6	7	8	9	10

6) Following treatment for this most recent relapse (attack, exacerbation), how much do you feel that you have returned to your baseline state of health without any residual relapse symptoms? *(Mark one)*

Got worse	No improvement	A Little			Somewhat			Very Much			Completely returned to baseline
☐	☐	☐	☐	☐	☐	☐	☐	☐	☐	☐	☐
	0	1	2	3	4	5	6	7	8	9	10

7) Did you have any side effects from the treatment you received for this relapse (attack, exacerbation)? *(Check all that apply)*

- ☐ Mood changes/depression/anxiety
- ☐ Weight gain
- ☐ Nausea and/or vomiting
- ☐ Sleep disturbance
- ☐ Increased blood pressure
- ☐ Low blood pressure
- ☐ Stomach upset or heartburn
- ☐ Headache
- ☐ Faintness (light headedness)
- ☐ High blood sugar
- ☐ Increased fatigue
- ☐ Dizziness
- ☐ Increased appetite
- ☐ Fever
- ☐ Muscle cramps
- ☐ Chills
- ☐ Infection
- ☐ Other:

If you have any questions, please ask your MS Nurse

For office use only

Date: _____________ Patient initials: ______ ______ ______

Type of MS (circle one): RRMS PPMS SPMS

Type of visit (circle one): Phone Office

Questionnaire completed by (circle one): Patient Office Staff

MS Relapse Evaluation—After Relapse Treatment *(1 month [± 1 week] follow up)*

Patient's age in years: ______

Patient's sex (circle one): Male Female

---

1) What treatment was prescribed for this most recent relapse (attack, exacerbation)? *(Check all that apply)*

☐ IV steroid infusion ☐ Oral steroid tablets (only) ☐ Oral steroid tablets (after IV steroids)
☐ Acthar/ACTH injections ☐ Plasma exchange ☐ No treatment
☐ I'm not sure ☐ Other: _______________

2) Did you complete the prescribed relapse treatment?

☐ Yes ☐ No If not, why? ____

3) How many days/months has it been since you completed treatment for this most recent relapse (attack, exacerbation)? _____

4) Do you think that the treatment for this most recent relapse (attack, exacerbation) resulted in improvement of your relapse symptoms? *(Mark one)*

Got worse	No improvement	A Little			Somewhat			Very Much			Completely resolved
☐	☐	☐	☐	☐	☐	☐	☐	☐	☐	☐	☐
	0	1	2	3	4	5	6	7	8	9	10

5) Following treatment for this most recent relapse (attack, exacerbation), how much are your relapse symptoms continuing to affect your daily activities or overall function? *(Mark one)*

Not at all	A Little			Somewhat			Very Much			Severely
☐	☐	☐	☐	☐	☐	☐	☐	☐	☐	☐
0	1	2	3	4	5	6	7	8	9	10

6) Following treatment for this most recent relapse (attack, exacerbation), how much do you feel that you have returned to your baseline state of health without any residual relapse symptoms? *(Mark one)*

Got worse	No improvement	A Little			Somewhat			Very Much			Completely returned to baseline
☐	☐	☐	☐	☐	☐	☐	☐	☐	☐	☐	☐
	0	1	2	3	4	5	6	7	8	9	10

7) Did you have any side effects from the treatment you received for this relapse (attack, exacerbation)? *(Check all that apply)*

☐ Mood changes/depression/anxiety ☐ Weight gain ☐ Nausea and/or vomiting
☐ Sleep disturbance ☐ Increased blood pressure ☐ Low blood pressure
☐ Stomach upset or heartburn ☐ Headache ☐ Faintness (light headedness)
☐ High blood sugar ☐ Increased fatigue ☐ Dizziness
☐ Increased appetite ☐ Fever ☐ Muscle cramps
☐ Chills ☐ Infection ☐ Other:

If you have any questions, please ask your MS Nurse

For office use only

Date: _____________ Patient initials: ______ ______ ______

Type of MS (circle one): RRMS PPMS SPMS

Type of visit (circle one): Phone Office

Questionnaire completed by (circle one): Patient Office Staff

MS Relapse Evaluation—After Relapse Treatment *(1 month [± 1 week] follow up)*

Patient's age in years: ______

Patient's sex (circle one): Male Female

---

1) What treatment was prescribed for this most recent relapse (attack, exacerbation)? *(Check all that apply)*

- ☐ IV steroid infusion
- ☐ Oral steroid tablets (only)
- ☐ Oral steroid tablets (after IV steroids)
- ☐ Acthar/ACTH injections
- ☐ Plasma exchange
- ☐ No treatment
- ☐ I'm not sure
- ☐ Other: _______________

2) Did you complete the prescribed relapse treatment?

☐ Yes ☐ No If not, why? ____

3) How many days/months has it been since you completed treatment for this most recent relapse (attack, exacerbation)? _____

4) Do you think that the treatment for this most recent relapse (attack, exacerbation) resulted in improvement of your relapse symptoms? *(Mark one)*

Got worse	No improvement	A Little			Somewhat			Very Much			Completely resolved
☐	☐	☐	☐	☐	☐	☐	☐	☐	☐	☐	☐
	0	1	2	3	4	5	6	7	8	9	10

5) Following treatment for this most recent relapse (attack, exacerbation), how much are your relapse symptoms continuing to affect your daily activities or overall function? *(Mark one)*

Not at all	A Little			Somewhat			Very Much			Severely
☐	☐	☐	☐	☐	☐	☐	☐	☐	☐	☐
0	1	2	3	4	5	6	7	8	9	10

6) Following treatment for this most recent relapse (attack, exacerbation), how much do you feel that you have returned to your baseline state of health without any residual relapse symptoms? *(Mark one)*

Got worse	No improvement	A Little			Somewhat			Very Much			Completely returned to baseline
☐	☐	☐	☐	☐	☐	☐	☐	☐	☐	☐	☐
	0	1	2	3	4	5	6	7	8	9	10

7) Did you have any side effects from the treatment you received for this relapse (attack, exacerbation)? *(Check all that apply)*

- ☐ Mood changes/depression/anxiety
- ☐ Sleep disturbance
- ☐ Stomach upset or heartburn
- ☐ High blood sugar
- ☐ Increased appetite
- ☐ Chills
- ☐ Weight gain
- ☐ Increased blood pressure
- ☐ Headache
- ☐ Increased fatigue
- ☐ Fever
- ☐ Infection
- ☐ Nausea and/or vomiting
- ☐ Low blood pressure
- ☐ Faintness (light headedness)
- ☐ Dizziness
- ☐ Muscle cramps
- ☐ Other:

If you have any questions, please ask your MS Nurse

For office use only

Date: _____________ Patient initials: ______ ______ ______

Type of MS (circle one): RRMS PPMS SPMS

Type of visit (circle one): Phone Office

Questionnaire completed by (circle one): Patient Office Staff

MS Relapse Evaluation—After Relapse Treatment *(1 month [± 1 week] follow up)*

Patient's age in years: ______

Patient's sex (circle one): Male Female

---

1) What treatment was prescribed for this most recent relapse (attack, exacerbation)? *(Check all that apply)*

- ☐ IV steroid infusion
- ☐ Oral steroid tablets (only)
- ☐ Oral steroid tablets (after IV steroids)
- ☐ Acthar/ACTH injections
- ☐ Plasma exchange
- ☐ No treatment
- ☐ I'm not sure
- ☐ Other: _______________

2) Did you complete the prescribed relapse treatment?

☐ Yes ☐ No If not, why? ____

3) How many days/months has it been since you completed treatment for this most recent relapse (attack, exacerbation)? _____

4) Do you think that the treatment for this most recent relapse (attack, exacerbation) resulted in improvement of your relapse symptoms? *(Mark one)*

Got worse	No improvement	A Little			Somewhat			Very Much			Completely resolved
☐	☐	☐	☐	☐	☐	☐	☐	☐	☐	☐	☐
	0	1	2	3	4	5	6	7	8	9	10

5) Following treatment for this most recent relapse (attack, exacerbation), how much are your relapse symptoms continuing to affect your daily activities or overall function? *(Mark one)*

Not at all	A Little			Somewhat			Very Much			Severely
☐	☐	☐	☐	☐	☐	☐	☐	☐	☐	☐
0	1	2	3	4	5	6	7	8	9	10

6) Following treatment for this most recent relapse (attack, exacerbation), how much do you feel that you have returned to your baseline state of health without any residual relapse symptoms? *(Mark one)*

Got worse	No improvement	A Little			Somewhat			Very Much			Completely returned to baseline
☐	☐	☐	☐	☐	☐	☐	☐	☐	☐	☐	☐
	0	1	2	3	4	5	6	7	8	9	10

7) Did you have any side effects from the treatment you received for this relapse (attack, exacerbation)? *(Check all that apply)*

- ☐ Mood changes/depression/anxiety
- ☐ Sleep disturbance
- ☐ Stomach upset or heartburn
- ☐ High blood sugar
- ☐ Increased appetite
- ☐ Chills
- ☐ Weight gain
- ☐ Increased blood pressure
- ☐ Headache
- ☐ Increased fatigue
- ☐ Fever
- ☐ Infection
- ☐ Nausea and/or vomiting
- ☐ Low blood pressure
- ☐ Faintness (light headedness)
- ☐ Dizziness
- ☐ Muscle cramps
- ☐ Other:

If you have any questions, please ask your MS Nurse

For office use only

Date: _____________ Patient initials: ______ ______ ______

Type of MS (circle one): RRMS PPMS SPMS

Type of visit (circle one): Phone Office

Questionnaire completed by (circle one): Patient Office Staff

MS Relapse Evaluation—After Relapse Treatment *(1 month [± 1 week] follow up)*

Patient's age in years: ______

Patient's sex (circle one): Male Female

---

1) What treatment was prescribed for this most recent relapse (attack, exacerbation)? *(Check all that apply)*

☐ IV steroid infusion ☐ Oral steroid tablets (only) ☐ Oral steroid tablets (after IV steroids)
☐ Acthar/ACTH injections ☐ Plasma exchange ☐ No treatment
☐ I'm not sure ☐ Other: ________________

2) Did you complete the prescribed relapse treatment?

☐ Yes ☐ No If not, why? ____

3) How many days/months has it been since you completed treatment for this most recent relapse (attack, exacerbation)? _____

4) Do you think that the treatment for this most recent relapse (attack, exacerbation) resulted in improvement of your relapse symptoms? *(Mark one)*

Got worse	No improvement	A Little			Somewhat			Very Much			Completely resolved
☐	☐	☐	☐	☐	☐	☐	☐	☐	☐	☐	☐
	0	1	2	3	4	5	6	7	8	9	10

5) Following treatment for this most recent relapse (attack, exacerbation), how much are your relapse symptoms continuing to affect your daily activities or overall function? *(Mark one)*

Not at all	A Little			Somewhat			Very Much			Severely
☐	☐	☐	☐	☐	☐	☐	☐	☐	☐	☐
0	1	2	3	4	5	6	7	8	9	10

6) Following treatment for this most recent relapse (attack, exacerbation), how much do you feel that you have returned to your baseline state of health without any residual relapse symptoms? *(Mark one)*

Got worse	No improvement	A Little			Somewhat			Very Much			Completely returned to baseline
☐	☐	☐	☐	☐	☐	☐	☐	☐	☐	☐	☐
	0	1	2	3	4	5	6	7	8	9	10

7) Did you have any side effects from the treatment you received for this relapse (attack, exacerbation)? *(Check all that apply)*

☐ Mood changes/depression/anxiety ☐ Weight gain ☐ Nausea and/or vomiting
☐ Sleep disturbance ☐ Increased blood pressure ☐ Low blood pressure
☐ Stomach upset or heartburn ☐ Headache ☐ Faintness (light headedness)
☐ High blood sugar ☐ Increased fatigue ☐ Dizziness
☐ Increased appetite ☐ Fever ☐ Muscle cramps
☐ Chills ☐ Infection ☐ Other:

If you have any questions, please ask your MS Nurse

For office use only

Date: _____________ Patient initials: ______ ______ ______

Type of MS (circle one): RRMS PPMS SPMS

Type of visit (circle one): Phone Office

Questionnaire completed by (circle one): Patient Office Staff

MS Relapse Evaluation—After Relapse Treatment *(1 month [± 1 week] follow up)*

Patient's age in years: ______

Patient's sex (circle one): Male Female

---

1) What treatment was prescribed for this most recent relapse (attack, exacerbation)? *(Check all that apply)*

- ☐ IV steroid infusion
- ☐ Oral steroid tablets (only)
- ☐ Oral steroid tablets (after IV steroids)
- ☐ Acthar/ACTH injections
- ☐ Plasma exchange
- ☐ No treatment
- ☐ I'm not sure
- ☐ Other: _______________

2) Did you complete the prescribed relapse treatment?

☐ Yes ☐ No If not, why? ____

3) How many days/months has it been since you completed treatment for this most recent relapse (attack, exacerbation)? _____

4) Do you think that the treatment for this most recent relapse (attack, exacerbation) resulted in improvement of your relapse symptoms? *(Mark one)*

Got worse	No improvement	A Little			Somewhat			Very Much			Completely resolved
☐	☐	☐	☐	☐	☐	☐	☐	☐	☐	☐	☐
	0	1	2	3	4	5	6	7	8	9	10

5) Following treatment for this most recent relapse (attack, exacerbation), how much are your relapse symptoms continuing to affect your daily activities or overall function? *(Mark one)*

Not at all	A Little			Somewhat			Very Much			Severely
☐	☐	☐	☐	☐	☐	☐	☐	☐	☐	☐
0	1	2	3	4	5	6	7	8	9	10

6) Following treatment for this most recent relapse (attack, exacerbation), how much do you feel that you have returned to your baseline state of health without any residual relapse symptoms? *(Mark one)*

Got worse	No improvement	A Little			Somewhat			Very Much			Completely returned to baseline
☐	☐	☐	☐	☐	☐	☐	☐	☐	☐	☐	☐
	0	1	2	3	4	5	6	7	8	9	10

7) Did you have any side effects from the treatment you received for this relapse (attack, exacerbation)? *(Check all that apply)*

- ☐ Mood changes/depression/anxiety
- ☐ Weight gain
- ☐ Nausea and/or vomiting
- ☐ Sleep disturbance
- ☐ Increased blood pressure
- ☐ Low blood pressure
- ☐ Stomach upset or heartburn
- ☐ Headache
- ☐ Faintness (light headedness)
- ☐ High blood sugar
- ☐ Increased fatigue
- ☐ Dizziness
- ☐ Increased appetite
- ☐ Fever
- ☐ Muscle cramps
- ☐ Chills
- ☐ Infection
- ☐ Other:

If you have any questions, please ask your MS Nurse

For office use only

Date: _____________ Patient initials: ______ ______ ______

Type of MS (circle one): RRMS PPMS SPMS

Type of visit (circle one): Phone Office

Questionnaire completed by (circle one): Patient Office Staff

MS Relapse Evaluation—After Relapse Treatment *(1 month [± 1 week] follow up)*

Patient's age in years: ______

Patient's sex (circle one): Male Female

---

1) What treatment was prescribed for this most recent relapse (attack, exacerbation)? *(Check all that apply)*

- ☐ IV steroid infusion
- ☐ Oral steroid tablets (only)
- ☐ Oral steroid tablets (after IV steroids)
- ☐ Acthar/ACTH injections
- ☐ Plasma exchange
- ☐ No treatment
- ☐ I'm not sure
- ☐ Other: _______________

2) Did you complete the prescribed relapse treatment?

☐ Yes ☐ No If not, why? ____

3) How many days/months has it been since you completed treatment for this most recent relapse (attack, exacerbation)? _____

4) Do you think that the treatment for this most recent relapse (attack, exacerbation) resulted in improvement of your relapse symptoms? *(Mark one)*

Got worse	No improvement	A Little			Somewhat			Very Much			Completely resolved
☐	☐	☐	☐	☐	☐	☐	☐	☐	☐	☐	☐
	0	1	2	3	4	5	6	7	8	9	10

5) Following treatment for this most recent relapse (attack, exacerbation), how much are your relapse symptoms continuing to affect your daily activities or overall function? *(Mark one)*

Not at all	A Little			Somewhat			Very Much			Severely
☐	☐	☐	☐	☐	☐	☐	☐	☐	☐	☐
0	1	2	3	4	5	6	7	8	9	10

6) Following treatment for this most recent relapse (attack, exacerbation), how much do you feel that you have returned to your baseline state of health without any residual relapse symptoms? *(Mark one)*

Got worse	No improvement	A Little			Somewhat			Very Much			Completely returned to baseline
☐	☐	☐	☐	☐	☐	☐	☐	☐	☐	☐	☐
	0	1	2	3	4	5	6	7	8	9	10

7) Did you have any side effects from the treatment you received for this relapse (attack, exacerbation)? *(Check all that apply)*

- ☐ Mood changes/depression/anxiety
- ☐ Sleep disturbance
- ☐ Stomach upset or heartburn
- ☐ High blood sugar
- ☐ Increased appetite
- ☐ Chills
- ☐ Weight gain
- ☐ Increased blood pressure
- ☐ Headache
- ☐ Increased fatigue
- ☐ Fever
- ☐ Infection
- ☐ Nausea and/or vomiting
- ☐ Low blood pressure
- ☐ Faintness (light headedness)
- ☐ Dizziness
- ☐ Muscle cramps
- ☐ Other:

If you have any questions, please ask your MS Nurse

For office use only

Date: _____________ Patient initials: ______ ______ ______

Type of MS (circle one): RRMS PPMS SPMS

Type of visit (circle one): Phone Office

Questionnaire completed by (circle one): Patient Office Staff

MS Relapse Evaluation—After Relapse Treatment *(1 month [± 1 week] follow up)*

Patient's age in years: ______

Patient's sex (circle one): Male Female

---

1) What treatment was prescribed for this most recent relapse (attack, exacerbation)? *(Check all that apply)*

- ☐ IV steroid infusion
- ☐ Oral steroid tablets (only)
- ☐ Oral steroid tablets (after IV steroids)
- ☐ Acthar/ACTH injections
- ☐ Plasma exchange
- ☐ No treatment
- ☐ I'm not sure
- ☐ Other: ________________

2) Did you complete the prescribed relapse treatment?

☐ Yes ☐ No If not, why? ____

3) How many days/months has it been since you completed treatment for this most recent relapse (attack, exacerbation)? _____

4) Do you think that the treatment for this most recent relapse (attack, exacerbation) resulted in improvement of your relapse symptoms? *(Mark one)*

Got worse	No improvement	A Little			Somewhat			Very Much			Completely resolved
☐	☐	☐	☐	☐	☐	☐	☐	☐	☐	☐	☐
	0	1	2	3	4	5	6	7	8	9	10

5) Following treatment for this most recent relapse (attack, exacerbation), how much are your relapse symptoms continuing to affect your daily activities or overall function? *(Mark one)*

Not at all	A Little			Somewhat			Very Much			Severely
☐	☐	☐	☐	☐	☐	☐	☐	☐	☐	☐
0	1	2	3	4	5	6	7	8	9	10

6) Following treatment for this most recent relapse (attack, exacerbation), how much do you feel that you have returned to your baseline state of health without any residual relapse symptoms? *(Mark one)*

Got worse	No improvement	A Little			Somewhat			Very Much			Completely returned to baseline
☐	☐	☐	☐	☐	☐	☐	☐	☐	☐	☐	☐
	0	1	2	3	4	5	6	7	8	9	10

7) Did you have any side effects from the treatment you received for this relapse (attack, exacerbation)? *(Check all that apply)*

- ☐ Mood changes/depression/anxiety
- ☐ Weight gain
- ☐ Nausea and/or vomiting
- ☐ Sleep disturbance
- ☐ Increased blood pressure
- ☐ Low blood pressure
- ☐ Stomach upset or heartburn
- ☐ Headache
- ☐ Faintness (light headedness)
- ☐ High blood sugar
- ☐ Increased fatigue
- ☐ Dizziness
- ☐ Increased appetite
- ☐ Fever
- ☐ Muscle cramps
- ☐ Chills
- ☐ Infection
- ☐ Other:

If you have any questions, please ask your MS Nurse

For office use only

Date: ______________ Patient initials: ______ ______ ______

Type of MS (circle one): RRMS PPMS SPMS

Type of visit (circle one): Phone Office

Questionnaire completed by (circle one): Patient Office Staff

MS Relapse Evaluation—After Relapse Treatment *(1 month [± 1 week] follow up)*

Patient's age in years: ______

Patient's sex (circle one): Male Female

---

1) What treatment was prescribed for this most recent relapse (attack, exacerbation)? *(Check all that apply)*

☐ IV steroid infusion ☐ Oral steroid tablets (only) ☐ Oral steroid tablets (after IV steroids)
☐ Acthar/ACTH injections ☐ Plasma exchange ☐ No treatment
☐ I'm not sure ☐ Other: _______________

2) Did you complete the prescribed relapse treatment?

☐ Yes ☐ No If not, why? ____

3) How many days/months has it been since you completed treatment for this most recent relapse (attack, exacerbation)? _____

4) Do you think that the treatment for this most recent relapse (attack, exacerbation) resulted in improvement of your relapse symptoms? *(Mark one)*

Got worse	No improvement	A Little			Somewhat			Very Much			Completely resolved
☐	☐	☐	☐	☐	☐	☐	☐	☐	☐	☐	☐
	0	1	2	3	4	5	6	7	8	9	10

5) Following treatment for this most recent relapse (attack, exacerbation), how much are your relapse symptoms continuing to affect your daily activities or overall function? *(Mark one)*

Not at all	A Little			Somewhat			Very Much			Severely
☐	☐	☐	☐	☐	☐	☐	☐	☐	☐	☐
0	1	2	3	4	5	6	7	8	9	10

6) Following treatment for this most recent relapse (attack, exacerbation), how much do you feel that you have returned to your baseline state of health without any residual relapse symptoms? *(Mark one)*

Got worse	No improvement	A Little			Somewhat			Very Much			Completely returned to baseline
☐	☐	☐	☐	☐	☐	☐	☐	☐	☐	☐	☐
	0	1	2	3	4	5	6	7	8	9	10

7) Did you have any side effects from the treatment you received for this relapse (attack, exacerbation)? *(Check all that apply)*

☐ Mood changes/depression/anxiety ☐ Weight gain ☐ Nausea and/or vomiting
☐ Sleep disturbance ☐ Increased blood pressure ☐ Low blood pressure
☐ Stomach upset or heartburn ☐ Headache ☐ Faintness (light headedness)
☐ High blood sugar ☐ Increased fatigue ☐ Dizziness
☐ Increased appetite ☐ Fever ☐ Muscle cramps
☐ Chills ☐ Infection ☐ Other:

If you have any questions, please ask your MS Nurse

For office use only

Date: _____________ Patient initials: ______ ______ ______

Type of MS (circle one): RRMS PPMS SPMS

Type of visit (circle one): Phone Office

Questionnaire completed by (circle one): Patient Office Staff

MS Relapse Evaluation—After Relapse Treatment *(1 month [± 1 week] follow up)*

Patient's age in years: ______

Patient's sex (circle one): Male Female

---

1) What treatment was prescribed for this most recent relapse (attack, exacerbation)? *(Check all that apply)*

- ☐ IV steroid infusion
- ☐ Oral steroid tablets (only)
- ☐ Oral steroid tablets (after IV steroids)
- ☐ Acthar/ACTH injections
- ☐ Plasma exchange
- ☐ No treatment
- ☐ I'm not sure
- ☐ Other: _______________

2) Did you complete the prescribed relapse treatment?

☐ Yes ☐ No If not, why? ____

3) How many days/months has it been since you completed treatment for this most recent relapse (attack, exacerbation)? _____

4) Do you think that the treatment for this most recent relapse (attack, exacerbation) resulted in improvement of your relapse symptoms? *(Mark one)*

Got worse	No improvement	A Little			Somewhat			Very Much			Completely resolved
☐	☐	☐	☐	☐	☐	☐	☐	☐	☐	☐	☐
	0	1	2	3	4	5	6	7	8	9	10

5) Following treatment for this most recent relapse (attack, exacerbation), how much are your relapse symptoms continuing to affect your daily activities or overall function? *(Mark one)*

Not at all	A Little			Somewhat			Very Much			Severely
☐	☐	☐	☐	☐	☐	☐	☐	☐	☐	☐
0	1	2	3	4	5	6	7	8	9	10

6) Following treatment for this most recent relapse (attack, exacerbation), how much do you feel that you have returned to your baseline state of health without any residual relapse symptoms? *(Mark one)*

Got worse	No improvement	A Little			Somewhat			Very Much			Completely returned to baseline
☐	☐	☐	☐	☐	☐	☐	☐	☐	☐	☐	☐
	0	1	2	3	4	5	6	7	8	9	10

7) Did you have any side effects from the treatment you received for this relapse (attack, exacerbation)? *(Check all that apply)*

- ☐ Mood changes/depression/anxiety
- ☐ Sleep disturbance
- ☐ Stomach upset or heartburn
- ☐ High blood sugar
- ☐ Increased appetite
- ☐ Chills
- ☐ Weight gain
- ☐ Increased blood pressure
- ☐ Headache
- ☐ Increased fatigue
- ☐ Fever
- ☐ Infection
- ☐ Nausea and/or vomiting
- ☐ Low blood pressure
- ☐ Faintness (light headedness)
- ☐ Dizziness
- ☐ Muscle cramps
- ☐ Other:

If you have any questions, please ask your MS Nurse

For office use only

Date: _____________ Patient initials: ______ ______ ______

Type of MS (circle one): RRMS PPMS SPMS

Type of visit (circle one): Phone Office

Questionnaire completed by (circle one): Patient Office Staff

MS Relapse Evaluation—After Relapse Treatment *(1 month [± 1 week] follow up)*

Patient's age in years: ______

Patient's sex (circle one): Male Female

---

1) What treatment was prescribed for this most recent relapse (attack, exacerbation)? *(Check all that apply)*

☐ IV steroid infusion ☐ Oral steroid tablets (only) ☐ Oral steroid tablets (after IV steroids)
☐ Acthar/ACTH injections ☐ Plasma exchange ☐ No treatment
☐ I'm not sure ☐ Other: _______________

2) Did you complete the prescribed relapse treatment?

☐ Yes ☐ No If not, why? ____

3) How many days/months has it been since you completed treatment for this most recent relapse (attack, exacerbation)? _____

4) Do you think that the treatment for this most recent relapse (attack, exacerbation) resulted in improvement of your relapse symptoms? *(Mark one)*

Got worse	No improvement	A Little			Somewhat			Very Much			Completely resolved
☐	☐	☐	☐	☐	☐	☐	☐	☐	☐	☐	☐
	0	1	2	3	4	5	6	7	8	9	10

5) Following treatment for this most recent relapse (attack, exacerbation), how much are your relapse symptoms continuing to affect your daily activities or overall function? *(Mark one)*

Not at all	A Little			Somewhat			Very Much			Severely
☐	☐	☐	☐	☐	☐	☐	☐	☐	☐	☐
0	1	2	3	4	5	6	7	8	9	10

6) Following treatment for this most recent relapse (attack, exacerbation), how much do you feel that you have returned to your baseline state of health without any residual relapse symptoms? *(Mark one)*

Got worse	No improvement	A Little			Somewhat			Very Much			Completely returned to baseline
☐	☐	☐	☐	☐	☐	☐	☐	☐	☐	☐	☐
	0	1	2	3	4	5	6	7	8	9	10

7) Did you have any side effects from the treatment you received for this relapse (attack, exacerbation)? *(Check all that apply)*

☐ Mood changes/depression/anxiety ☐ Weight gain ☐ Nausea and/or vomiting
☐ Sleep disturbance ☐ Increased blood pressure ☐ Low blood pressure
☐ Stomach upset or heartburn ☐ Headache ☐ Faintness (light headedness)
☐ High blood sugar ☐ Increased fatigue ☐ Dizziness
☐ Increased appetite ☐ Fever ☐ Muscle cramps
☐ Chills ☐ Infection ☐ Other:

If you have any questions, please ask your MS Nurse

For office use only

Date: _____________ Patient initials: ______ ______ ______

Type of MS (circle one): RRMS PPMS SPMS

Type of visit (circle one): Phone Office

Questionnaire completed by (circle one): Patient Office Staff

MS Relapse Evaluation—After Relapse Treatment *(1 month [± 1 week] follow up)*

Patient's age in years: ______

Patient's sex (circle one): Male Female

---

1) What treatment was prescribed for this most recent relapse (attack, exacerbation)? *(Check all that apply)*

☐ IV steroid infusion ☐ Oral steroid tablets (only) ☐ Oral steroid tablets (after IV steroids)
☐ Acthar/ACTH injections ☐ Plasma exchange ☐ No treatment
☐ I'm not sure ☐ Other: ________________

2) Did you complete the prescribed relapse treatment?
☐ Yes ☐ No If not, why? ____

3) How many days/months has it been since you completed treatment for this most recent relapse (attack, exacerbation)? _____

4) Do you think that the treatment for this most recent relapse (attack, exacerbation) resulted in improvement of your relapse symptoms? *(Mark one)*

Got worse	No improvement	A Little			Somewhat			Very Much			Completely resolved
☐	☐	☐	☐	☐	☐	☐	☐	☐	☐	☐	☐
	0	1	2	3	4	5	6	7	8	9	10

5) Following treatment for this most recent relapse (attack, exacerbation), how much are your relapse symptoms continuing to affect your daily activities or overall function? *(Mark one)*

Not at all	A Little			Somewhat			Very Much			Severely
☐	☐	☐	☐	☐	☐	☐	☐	☐	☐	☐
0	1	2	3	4	5	6	7	8	9	10

6) Following treatment for this most recent relapse (attack, exacerbation), how much do you feel that you have returned to your baseline state of health without any residual relapse symptoms? *(Mark one)*

Got worse	No improvement	A Little			Somewhat			Very Much			Completely returned to baseline
☐	☐	☐	☐	☐	☐	☐	☐	☐	☐	☐	☐
	0	1	2	3	4	5	6	7	8	9	10

7) Did you have any side effects from the treatment you received for this relapse (attack, exacerbation)? *(Check all that apply)*

☐ Mood changes/depression/anxiety ☐ Weight gain ☐ Nausea and/or vomiting
☐ Sleep disturbance ☐ Increased blood pressure ☐ Low blood pressure
☐ Stomach upset or heartburn ☐ Headache ☐ Faintness (light headedness)
☐ High blood sugar ☐ Increased fatigue ☐ Dizziness
☐ Increased appetite ☐ Fever ☐ Muscle cramps
☐ Chills ☐ Infection ☐ Other:

If you have any questions, please ask your MS Nurse

For office use only

Date: ______________ Patient initials: ______ ______ ______

Type of MS (circle one): RRMS PPMS SPMS

Type of visit (circle one): Phone Office

Questionnaire completed by (circle one): Patient Office Staff

MS Relapse Evaluation—After Relapse Treatment *(1 month [± 1 week] follow up)*

Patient's age in years: ______

Patient's sex (circle one): Male Female

---

1) What treatment was prescribed for this most recent relapse (attack, exacerbation)? *(Check all that apply)*

- ☐ IV steroid infusion
- ☐ Oral steroid tablets (only)
- ☐ Oral steroid tablets (after IV steroids)
- ☐ Acthar/ACTH injections
- ☐ Plasma exchange
- ☐ No treatment
- ☐ I'm not sure
- ☐ Other: _______________

2) Did you complete the prescribed relapse treatment?

☐ Yes ☐ No If not, why? ____

3) How many days/months has it been since you completed treatment for this most recent relapse (attack, exacerbation)? _____

4) Do you think that the treatment for this most recent relapse (attack, exacerbation) resulted in improvement of your relapse symptoms? *(Mark one)*

Got worse	No improvement	A Little			Somewhat			Very Much			Completely resolved
☐	☐	☐	☐	☐	☐	☐	☐	☐	☐	☐	☐
	0	1	2	3	4	5	6	7	8	9	10

5) Following treatment for this most recent relapse (attack, exacerbation), how much are your relapse symptoms continuing to affect your daily activities or overall function? *(Mark one)*

Not at all	A Little			Somewhat			Very Much			Severely
☐	☐	☐	☐	☐	☐	☐	☐	☐	☐	☐
0	1	2	3	4	5	6	7	8	9	10

6) Following treatment for this most recent relapse (attack, exacerbation), how much do you feel that you have returned to your baseline state of health without any residual relapse symptoms? *(Mark one)*

Got worse	No improvement	A Little			Somewhat			Very Much			Completely returned to baseline
☐	☐	☐	☐	☐	☐	☐	☐	☐	☐	☐	☐
	0	1	2	3	4	5	6	7	8	9	10

7) Did you have any side effects from the treatment you received for this relapse (attack, exacerbation)? *(Check all that apply)*

- ☐ Mood changes/depression/anxiety
- ☐ Sleep disturbance
- ☐ Stomach upset or heartburn
- ☐ High blood sugar
- ☐ Increased appetite
- ☐ Chills
- ☐ Weight gain
- ☐ Increased blood pressure
- ☐ Headache
- ☐ Increased fatigue
- ☐ Fever
- ☐ Infection
- ☐ Nausea and/or vomiting
- ☐ Low blood pressure
- ☐ Faintness (light headedness)
- ☐ Dizziness
- ☐ Muscle cramps
- ☐ Other:

If you have any questions, please ask your MS Nurse

For office use only

Date: _____________ Patient initials: ______ ______ ______

Type of MS (circle one): RRMS PPMS SPMS

Type of visit (circle one): Phone Office

Questionnaire completed by (circle one): Patient Office Staff

MS Relapse Evaluation—After Relapse Treatment *(1 month [± 1 week] follow up)*

Patient's age in years: ______

Patient's sex (circle one): Male Female

---

1) What treatment was prescribed for this most recent relapse (attack, exacerbation)? *(Check all that apply)*

☐ IV steroid infusion ☐ Oral steroid tablets (only) ☐ Oral steroid tablets (after IV steroids)
☐ Acthar/ACTH injections ☐ Plasma exchange ☐ No treatment
☐ I'm not sure ☐ Other: _______________

2) Did you complete the prescribed relapse treatment?

☐ Yes ☐ No If not, why? ________________________________

3) How many days/months has it been since you completed treatment for this most recent relapse (attack, exacerbation)? _____

4) Do you think that the treatment for this most recent relapse (attack, exacerbation) resulted in improvement of your relapse symptoms? *(Mark one)*

Got worse	No improvement	A Little			Somewhat			Very Much			Completely resolved
☐	☐	☐	☐	☐	☐	☐	☐	☐	☐	☐	☐
	0	1	2	3	4	5	6	7	8	9	10

5) Following treatment for this most recent relapse (attack, exacerbation), how much are your relapse symptoms continuing to affect your daily activities or overall function? *(Mark one)*

Not at all	A Little			Somewhat			Very Much			Severely
☐	☐	☐	☐	☐	☐	☐	☐	☐	☐	☐
0	1	2	3	4	5	6	7	8	9	10

6) Following treatment for this most recent relapse (attack, exacerbation), how much do you feel that you have returned to your baseline state of health without any residual relapse symptoms? *(Mark one)*

Got worse	No improvement	A Little			Somewhat			Very Much			Completely returned to baseline
☐	☐	☐	☐	☐	☐	☐	☐	☐	☐	☐	☐
	0	1	2	3	4	5	6	7	8	9	10

7) Did you have any side effects from the treatment you received for this relapse (attack, exacerbation)? *(Check all that apply)*

☐ Mood changes/depression/anxiety ☐ Weight gain ☐ Nausea and/or vomiting
☐ Sleep disturbance ☐ Increased blood pressure ☐ Low blood pressure
☐ Stomach upset or heartburn ☐ Headache ☐ Faintness (light headedness)
☐ High blood sugar ☐ Increased fatigue ☐ Dizziness
☐ Increased appetite ☐ Fever ☐ Muscle cramps
☐ Chills ☐ Infection ☐ Other:

If you have any questions, please ask your MS Nurse

For office use only

Date: _____________ Patient initials: ______ ______ ______

Type of MS (circle one): RRMS PPMS SPMS

Type of visit (circle one): Phone Office

Questionnaire completed by (circle one): Patient Office Staff

MS Relapse Evaluation—After Relapse Treatment *(1 month [± 1 week] follow up)*

Patient's age in years: ______

Patient's sex (circle one): Male Female

---

1) What treatment was prescribed for this most recent relapse (attack, exacerbation)? *(Check all that apply)*

☐ IV steroid infusion ☐ Oral steroid tablets (only) ☐ Oral steroid tablets (after IV steroids)
☐ Acthar/ACTH injections ☐ Plasma exchange ☐ No treatment
☐ I'm not sure ☐ Other: _______________

2) Did you complete the prescribed relapse treatment?

☐ Yes ☐ No If not, why? ____

3) How many days/months has it been since you completed treatment for this most recent relapse (attack, exacerbation)? _____

4) Do you think that the treatment for this most recent relapse (attack, exacerbation) resulted in improvement of your relapse symptoms? *(Mark one)*

Got worse	No improvement	A Little			Somewhat			Very Much			Completely resolved
☐	☐	☐	☐	☐	☐	☐	☐	☐	☐	☐	☐
	0	1	2	3	4	5	6	7	8	9	10

5) Following treatment for this most recent relapse (attack, exacerbation), how much are your relapse symptoms continuing to affect your daily activities or overall function? *(Mark one)*

Not at all	A Little			Somewhat			Very Much			Severely
☐	☐	☐	☐	☐	☐	☐	☐	☐	☐	☐
0	1	2	3	4	5	6	7	8	9	10

6) Following treatment for this most recent relapse (attack, exacerbation), how much do you feel that you have returned to your baseline state of health without any residual relapse symptoms? *(Mark one)*

Got worse	No improvement	A Little			Somewhat			Very Much			Completely returned to baseline
☐	☐	☐	☐	☐	☐	☐	☐	☐	☐	☐	☐
	0	1	2	3	4	5	6	7	8	9	10

7) Did you have any side effects from the treatment you received for this relapse (attack, exacerbation)? *(Check all that apply)*

☐ Mood changes/depression/anxiety ☐ Weight gain ☐ Nausea and/or vomiting
☐ Sleep disturbance ☐ Increased blood pressure ☐ Low blood pressure
☐ Stomach upset or heartburn ☐ Headache ☐ Faintness (light headedness)
☐ High blood sugar ☐ Increased fatigue ☐ Dizziness
☐ Increased appetite ☐ Fever ☐ Muscle cramps
☐ Chills ☐ Infection ☐ Other:

If you have any questions, please ask your MS Nurse

For office use only

Date: _____________ Patient initials: ______ ______ ______

Type of MS (circle one): RRMS PPMS SPMS

Type of visit (circle one): Phone Office

Questionnaire completed by (circle one): Patient Office Staff

MS Relapse Evaluation—After Relapse Treatment *(1 month [± 1 week] follow up)*

Patient's age in years: ______

Patient's sex (circle one): Male Female

---

1) What treatment was prescribed for this most recent relapse (attack, exacerbation)? *(Check all that apply)*

☐ IV steroid infusion ☐ Oral steroid tablets (only) ☐ Oral steroid tablets (after IV steroids)
☐ Acthar/ACTH injections ☐ Plasma exchange ☐ No treatment
☐ I'm not sure ☐ Other: _______________

2) Did you complete the prescribed relapse treatment?

☐ Yes ☐ No If not, why? ____

3) How many days/months has it been since you completed treatment for this most recent relapse (attack, exacerbation)? _____

4) Do you think that the treatment for this most recent relapse (attack, exacerbation) resulted in improvement of your relapse symptoms? *(Mark one)*

Got worse	No improvement	A Little			Somewhat			Very Much			Completely resolved
☐	☐	☐	☐	☐	☐	☐	☐	☐	☐	☐	☐
	0	1	2	3	4	5	6	7	8	9	10

5) Following treatment for this most recent relapse (attack, exacerbation), how much are your relapse symptoms continuing to affect your daily activities or overall function? *(Mark one)*

Not at all	A Little			Somewhat			Very Much			Severely
☐	☐	☐	☐	☐	☐	☐	☐	☐	☐	☐
0	1	2	3	4	5	6	7	8	9	10

6) Following treatment for this most recent relapse (attack, exacerbation), how much do you feel that you have returned to your baseline state of health without any residual relapse symptoms? *(Mark one)*

Got worse	No improvement	A Little			Somewhat			Very Much			Completely returned to baseline
☐	☐	☐	☐	☐	☐	☐	☐	☐	☐	☐	☐
	0	1	2	3	4	5	6	7	8	9	10

7) Did you have any side effects from the treatment you received for this relapse (attack, exacerbation)? *(Check all that apply)*

☐ Mood changes/depression/anxiety ☐ Weight gain ☐ Nausea and/or vomiting
☐ Sleep disturbance ☐ Increased blood pressure ☐ Low blood pressure
☐ Stomach upset or heartburn ☐ Headache ☐ Faintness (light headedness)
☐ High blood sugar ☐ Increased fatigue ☐ Dizziness
☐ Increased appetite ☐ Fever ☐ Muscle cramps
☐ Chills ☐ Infection ☐ Other:

If you have any questions, please ask your MS Nurse

For office use only

Date: _____________ Patient initials: ______ ______ ______

Type of MS (circle one): RRMS PPMS SPMS

Type of visit (circle one): Phone Office

Questionnaire completed by (circle one): Patient Office Staff

MS Relapse Evaluation—After Relapse Treatment *(1 month [± 1 week] follow up)*

Patient's age in years: ______

Patient's sex (circle one): Male Female

---

1) What treatment was prescribed for this most recent relapse (attack, exacerbation)? *(Check all that apply)*

- ☐ IV steroid infusion
- ☐ Oral steroid tablets (only)
- ☐ Oral steroid tablets (after IV steroids)
- ☐ Acthar/ACTH injections
- ☐ Plasma exchange
- ☐ No treatment
- ☐ I'm not sure
- ☐ Other: _______________

2) Did you complete the prescribed relapse treatment?

☐ Yes ☐ No If not, why? ____

3) How many days/months has it been since you completed treatment for this most recent relapse (attack, exacerbation)? _____

4) Do you think that the treatment for this most recent relapse (attack, exacerbation) resulted in improvement of your relapse symptoms? *(Mark one)*

Got worse	No improvement	A Little			Somewhat			Very Much			Completely resolved
☐	☐	☐	☐	☐	☐	☐	☐	☐	☐	☐	☐
	0	1	2	3	4	5	6	7	8	9	10

5) Following treatment for this most recent relapse (attack, exacerbation), how much are your relapse symptoms continuing to affect your daily activities or overall function? *(Mark one)*

Not at all	A Little			Somewhat			Very Much			Severely
☐	☐	☐	☐	☐	☐	☐	☐	☐	☐	☐
0	1	2	3	4	5	6	7	8	9	10

6) Following treatment for this most recent relapse (attack, exacerbation), how much do you feel that you have returned to your baseline state of health without any residual relapse symptoms? *(Mark one)*

Got worse	No improvement	A Little			Somewhat			Very Much			Completely returned to baseline
☐	☐	☐	☐	☐	☐	☐	☐	☐	☐	☐	☐
	0	1	2	3	4	5	6	7	8	9	10

7) Did you have any side effects from the treatment you received for this relapse (attack, exacerbation)? *(Check all that apply)*

- ☐ Mood changes/depression/anxiety
- ☐ Weight gain
- ☐ Nausea and/or vomiting
- ☐ Sleep disturbance
- ☐ Increased blood pressure
- ☐ Low blood pressure
- ☐ Stomach upset or heartburn
- ☐ Headache
- ☐ Faintness (light headedness)
- ☐ High blood sugar
- ☐ Increased fatigue
- ☐ Dizziness
- ☐ Increased appetite
- ☐ Fever
- ☐ Muscle cramps
- ☐ Chills
- ☐ Infection
- ☐ Other:

If you have any questions, please ask your MS Nurse

For office use only

Date: _____________ Patient initials: ______ ______ ______

Type of MS (circle one): RRMS PPMS SPMS

Type of visit (circle one): Phone Office

Questionnaire completed by (circle one): Patient Office Staff

MS Relapse Evaluation—After Relapse Treatment *(1 month [± 1 week] follow up)*

Patient's age in years: ______

Patient's sex (circle one): Male Female

1) What treatment was prescribed for this most recent relapse (attack, exacerbation)? *(Check all that apply)*
   - ☐ IV steroid infusion
   - ☐ Oral steroid tablets (only)
   - ☐ Oral steroid tablets (after IV steroids)
   - ☐ Acthar/ACTH injections
   - ☐ Plasma exchange
   - ☐ No treatment
   - ☐ I'm not sure
   - ☐ Other: _______________

2) Did you complete the prescribed relapse treatment?
   ☐ Yes ☐ No If not, why? ____________________

3) How many days/months has it been since you completed treatment for this most recent relapse (attack, exacerbation)? _____

4) Do you think that the treatment for this most recent relapse (attack, exacerbation) resulted in improvement of your relapse symptoms? *(Mark one)*

Got worse	No improvement	A Little			Somewhat			Very Much			Completely resolved
☐	☐	☐	☐	☐	☐	☐	☐	☐	☐	☐	☐
	0	1	2	3	4	5	6	7	8	9	10

5) Following treatment for this most recent relapse (attack, exacerbation), how much are your relapse symptoms continuing to affect your daily activities or overall function? *(Mark one)*

Not at all	A Little			Somewhat			Very Much			Severely
☐	☐	☐	☐	☐	☐	☐	☐	☐	☐	☐
0	1	2	3	4	5	6	7	8	9	10

6) Following treatment for this most recent relapse (attack, exacerbation), how much do you feel that you have returned to your baseline state of health without any residual relapse symptoms? *(Mark one)*

Got worse	No improvement	A Little			Somewhat			Very Much			Completely returned to baseline
☐	☐	☐	☐	☐	☐	☐	☐	☐	☐	☐	☐
	0	1	2	3	4	5	6	7	8	9	10

7) Did you have any side effects from the treatment you received for this relapse (attack, exacerbation)? *(Check all that apply)*
   - ☐ Mood changes/depression/anxiety
   - ☐ Weight gain
   - ☐ Nausea and/or vomiting
   - ☐ Sleep disturbance
   - ☐ Increased blood pressure
   - ☐ Low blood pressure
   - ☐ Stomach upset or heartburn
   - ☐ Headache
   - ☐ Faintness (light headedness)
   - ☐ High blood sugar
   - ☐ Increased fatigue
   - ☐ Dizziness
   - ☐ Increased appetite
   - ☐ Fever
   - ☐ Muscle cramps
   - ☐ Chills
   - ☐ Infection
   - ☐ Other:

If you have any questions, please ask your MS Nurse

For office use only

Date: _____________ Patient initials: ______ ______ ______

Type of MS (circle one): RRMS PPMS SPMS

Type of visit (circle one): Phone Office

Questionnaire completed by (circle one): Patient Office Staff

MS Relapse Evaluation—After Relapse Treatment *(1 month [± 1 week] follow up)*

Patient's age in years: ______

Patient's sex (circle one): Male Female

---

1) What treatment was prescribed for this most recent relapse (attack, exacerbation)? *(Check all that apply)*

- ☐ IV steroid infusion
- ☐ Oral steroid tablets (only)
- ☐ Oral steroid tablets (after IV steroids)
- ☐ Acthar/ACTH injections
- ☐ Plasma exchange
- ☐ No treatment
- ☐ I'm not sure
- ☐ Other: _______________

2) Did you complete the prescribed relapse treatment?

☐ Yes ☐ No If not, why? ____

3) How many days/months has it been since you completed treatment for this most recent relapse (attack, exacerbation)? _____

4) Do you think that the treatment for this most recent relapse (attack, exacerbation) resulted in improvement of your relapse symptoms? *(Mark one)*

Got worse	No improvement	A Little			Somewhat			Very Much			Completely resolved
☐	☐	☐	☐	☐	☐	☐	☐	☐	☐	☐	☐
	0	1	2	3	4	5	6	7	8	9	10

5) Following treatment for this most recent relapse (attack, exacerbation), how much are your relapse symptoms continuing to affect your daily activities or overall function? *(Mark one)*

Not at all	A Little			Somewhat			Very Much			Severely
☐	☐	☐	☐	☐	☐	☐	☐	☐	☐	☐
0	1	2	3	4	5	6	7	8	9	10

6) Following treatment for this most recent relapse (attack, exacerbation), how much do you feel that you have returned to your baseline state of health without any residual relapse symptoms? *(Mark one)*

Got worse	No improvement	A Little			Somewhat			Very Much			Completely returned to baseline
☐	☐	☐	☐	☐	☐	☐	☐	☐	☐	☐	☐
	0	1	2	3	4	5	6	7	8	9	10

7) Did you have any side effects from the treatment you received for this relapse (attack, exacerbation)? *(Check all that apply)*

- ☐ Mood changes/depression/anxiety
- ☐ Sleep disturbance
- ☐ Stomach upset or heartburn
- ☐ High blood sugar
- ☐ Increased appetite
- ☐ Chills
- ☐ Weight gain
- ☐ Increased blood pressure
- ☐ Headache
- ☐ Increased fatigue
- ☐ Fever
- ☐ Infection
- ☐ Nausea and/or vomiting
- ☐ Low blood pressure
- ☐ Faintness (light headedness)
- ☐ Dizziness
- ☐ Muscle cramps
- ☐ Other:

If you have any questions, please ask your MS Nurse

For office use only

Date: _____________ Patient initials: ______ ______ ______

Type of MS (circle one): RRMS PPMS SPMS

Type of visit (circle one): Phone Office

Questionnaire completed by (circle one): Patient Office Staff

MS Relapse Evaluation—After Relapse Treatment *(1 month [± 1 week] follow up)*

Patient's age in years: ______

Patient's sex (circle one): Male Female

---

1) What treatment was prescribed for this most recent relapse (attack, exacerbation)? *(Check all that apply)*

- ☐ IV steroid infusion
- ☐ Oral steroid tablets (only)
- ☐ Oral steroid tablets (after IV steroids)
- ☐ Acthar/ACTH injections
- ☐ Plasma exchange
- ☐ No treatment
- ☐ I'm not sure
- ☐ Other: _______________

2) Did you complete the prescribed relapse treatment?

☐ Yes ☐ No If not, why? ____

3) How many days/months has it been since you completed treatment for this most recent relapse (attack, exacerbation)? _____

4) Do you think that the treatment for this most recent relapse (attack, exacerbation) resulted in improvement of your relapse symptoms? *(Mark one)*

Got worse	No improvement	A Little			Somewhat			Very Much			Completely resolved
☐	☐	☐	☐	☐	☐	☐	☐	☐	☐	☐	☐
	0	1	2	3	4	5	6	7	8	9	10

5) Following treatment for this most recent relapse (attack, exacerbation), how much are your relapse symptoms continuing to affect your daily activities or overall function? *(Mark one)*

Not at all	A Little			Somewhat			Very Much			Severely
☐	☐	☐	☐	☐	☐	☐	☐	☐	☐	☐
0	1	2	3	4	5	6	7	8	9	10

6) Following treatment for this most recent relapse (attack, exacerbation), how much do you feel that you have returned to your baseline state of health without any residual relapse symptoms? *(Mark one)*

Got worse	No improvement	A Little			Somewhat			Very Much			Completely returned to baseline
☐	☐	☐	☐	☐	☐	☐	☐	☐	☐	☐	☐
	0	1	2	3	4	5	6	7	8	9	10

7) Did you have any side effects from the treatment you received for this relapse (attack, exacerbation)? *(Check all that apply)*

- ☐ Mood changes/depression/anxiety
- ☐ Weight gain
- ☐ Nausea and/or vomiting
- ☐ Sleep disturbance
- ☐ Increased blood pressure
- ☐ Low blood pressure
- ☐ Stomach upset or heartburn
- ☐ Headache
- ☐ Faintness (light headedness)
- ☐ High blood sugar
- ☐ Increased fatigue
- ☐ Dizziness
- ☐ Increased appetite
- ☐ Fever
- ☐ Muscle cramps
- ☐ Chills
- ☐ Infection
- ☐ Other:

If you have any questions, please ask your MS Nurse

For office use only

Date: _____________ Patient initials: ______ ______ ______

Type of MS (circle one): RRMS PPMS SPMS

Type of visit (circle one): Phone Office

Questionnaire completed by (circle one): Patient Office Staff
